# Suppressing mechanical property variability in recycled plastics via bioinspired design

**DOI:** 10.1073/pnas.2502613122

**Published:** 2025-08-12

**Authors:** Dimitrios Georgiou, Danqi Sun, Xing Liu, Christos E. Athanasiou

**Affiliations:** ^a^Daniel Guggenheim School of Aerospace Engineering, Georgia Institute of Technology, Atlanta, GA 30332; ^b^Department of Mechanical and Industrial Engineering, New Jersey Institute of Technology, Newark, NJ 07102

**Keywords:** recycled plastics, mechanical property variability, bioinspired design, tension-shear-chain model, uncertainty in material properties

## Abstract

Over 350 million metric tons of plastic waste are generated annually, with most ending up in landfills, dumps, or the environment, posing significant risks. Mechanical recycling remains underutilized, largely due to the high variability in the mechanical properties of recycled plastics (recyclates). This variability undermines performance reliability and hinders the adoption of recyclates in demanding industrial applications. Inspired by natural materials, known for their mechanical robustness despite microstructural stochasticity, we propose a universal, chemistry-agnostic, brick-and-mortar design tailored for recycled polymers. In this design, stiff recycled plastic platelets (bricks) are embedded in a soft virgin polymer matrix (mortar), which accommodates deformation and redistributes stress. To predict the effective modulus, strength, and property variability of such structures, we developed an uncertainty-aware tension-shear-chain model, combining Monte Carlo simulations with literature-based distributions of recyclates’ stiffness and conservative interfacial parameter stochasticity assumptions. We validated our model using nacre-inspired composites fabricated from recycled high-density polyethylene (rHDPE) platelets and polydimethylsiloxane (PDMS) mortar. The experimental results matched model predictions, confirming significant suppression of variability. In a case study on industrial HDPE stretch film, our design reduced modulus variability by up to 93% and maximum permissible strain variability by at least 68% compared to input rHDPE, while matching the modulus of virgin HDPE film. This work introduces a design-enabled variability-suppression strategy for recycled plastics, able to transform highly heterogenous materials into structurally robust products. By supporting virgin-plastic substitution and circular design strategies, our approach can enable the broader adoption of recyclates by several industries.

## Significance

Recycled plastics are key for circular economies, yet their widespread adoption is limited by unpredictable mechanical performance. This variability discourages their adoption, particularly in demanding applications with tight specifications. Inspired by natural materials like nacre, we present a brick-and-mortar composite design that dramatically suppresses property variability by embedding recycled polymer platelets in a soft matrix. Combined with an uncertainty-aware modeling framework validated experimentally, this approach reduces modulus variability by over 90% while matching the performance of virgin materials. Our design introduces a universally applicable, chemistry-agnostic solution that can enable the design of robust structures from materials exhibiting stochastic mechanical performance, thus allowing for the reliable use of recycled plastics in demanding applications, contributing to the global plastic waste problem.

Over 400 million metric tons of plastics are produced annually, with this amount projected to reach 1 billion tons per year by 2050 (1, 2). Each year, more than 350 million metric tons of plastic waste is generated, with the majority of this waste (about 70%) accumulating in landfills, dumps, and the natural environment (3), and posing significant environmental and health risks (4). Recycling plastic waste is necessary to address these issues and eventually transition away from using virgin, fossil-based resources for making materials.

Mechanical recycling, i.e., shredding, melting, and reforming plastic waste, is currently the most resource-efficient plastics recycling option (5). However, mechanically recycled plastics (recyclates) often exhibit a huge variability in their mechanical performance resulting in inconsistent quality compared to their virgin counterparts. There is consensus in the literature that heterogeneities and compositional fluctuation are inherent features of recyclates. For example, La Mantia and Vinci observed the erratic fracture behavior of postconsumer recycled polyethylene terephthalate (rPET) bottles under dry and humid conditions across multiple recycling cycles (6). Eriksen et al. observed significant variability in the strength and tensile strain of recycled high-density polyethylene (rHDPE) and recycled low-density polyethylene (rLDPE) sourced from household waste, ranging from 22 to 50 MPa and 11 to 18%, respectively, attributing it to the heterogeneity of the waste stream (7). Cecon et al. evaluated the performance of mixed plastic waste sourced from U.S. material recovery facilities (MRFs) and observed significant variability in the Young’s modulus across different MRFs, with the highest variability reported for HDPE ranging from 380 to 1,250 MPa (8).

The variability in the mechanical properties of recyclates, which arises even when the same polymer types are used as feedstocks (9), significantly limits their adoption by manufacturing industries in applications that require predictable, high-performance materials, due to concerns over inconsistent quality and performance reliability. While recycled polymers such as rPET or rHDPE can, at times, meet or even exceed the required mechanical specifications (e.g., tensile strength, elongation at break) (10), they often do so with high variability across batches, stemming from compositional fluctuations, feedstock contamination during the recycling process, degradation history, and processing conditions (7, 11, 12). This inconsistency introduces quality control risks and complicates compliance with stringent product standards, especially in applications demanding tight tolerances, such as food-grade packaging, pressure piping, or structural components (13, 14). Hence, recyclates are not used in these applications.

Notably, despite these variability challenges, recycled plastics are already used at scale in a wide range of commercial products. For example, rPET is commonly used in beverage bottles and textile fibers, while rHDPE is often incorporated into containers, pipes, or automotive parts—typically in noncritical sections or with a capped percentage of recycled content. In most of these cases, manufacturers adjust formulations or performance expectations to accommodate variability. Nevertheless, when performance specifications are strict or mechanical consistency is paramount, manufacturers tend to favor virgin polymers with predictable properties and certified performance. Thus, reducing variability in recyclates remains a critical enabling step toward expanding their use in more demanding applications and improving the reliability of recycled materials in the broader circular economy (9).

Several approaches have been introduced to address this issue focusing mainly on improving sorting (15), pretreatment, and process control (12, 16–18). However, applying these strategies at scale faces significant limitations. Sorting technologies often struggle to achieve sufficient material purity, especially in cases of multilayered films or mixed-material plastics (19). Pretreatment methods such as washing or decontamination can increase costs and still fail to remove deeply embedded additives or legacy contaminants (14, 20). Process control techniques, e.g., closed-loop injection molding (21), in-line melt rheometry (22), and real-time spectroscopic analysis (23) can adjust processing parameters on the fly to compensate for feedstock variability, but face high capital costs, sensor drifts, calibration demands, feedback latency, and spectral overlap. As a result, process control improvements during reprocessing help to stabilize output but cannot fully compensate for upstream material inconsistencies. Therefore, the fundamental challenge of transforming recyclates with stochastic properties into product of consistent quality persists.

Interestingly, natural materials, such as nacre and bamboo, do not exhibit significant variability in their mechanical performance and properties despite the compositional fluctuations and impurities introduced during their lifecycle (24–27). This is due to their brick-and-mortar architecture, which combines stiff mineral platelets with thin proteinic layers, exhibiting remarkable insensitivity to flaws at the nanoscale and thus extraordinary consistency in their fracture resistance (28). This hierarchical architecture employs multiple energy-dissipation mechanisms, such as crack deflection, tile interlocking, and crack bridging, to thwart catastrophic failure, while the proteinaceous layers accommodate plastic deformation and redistribute stress, to ensure mechanical robustness despite microscopic defects and microstructural randomness (29–34).

**Fig. 1.**
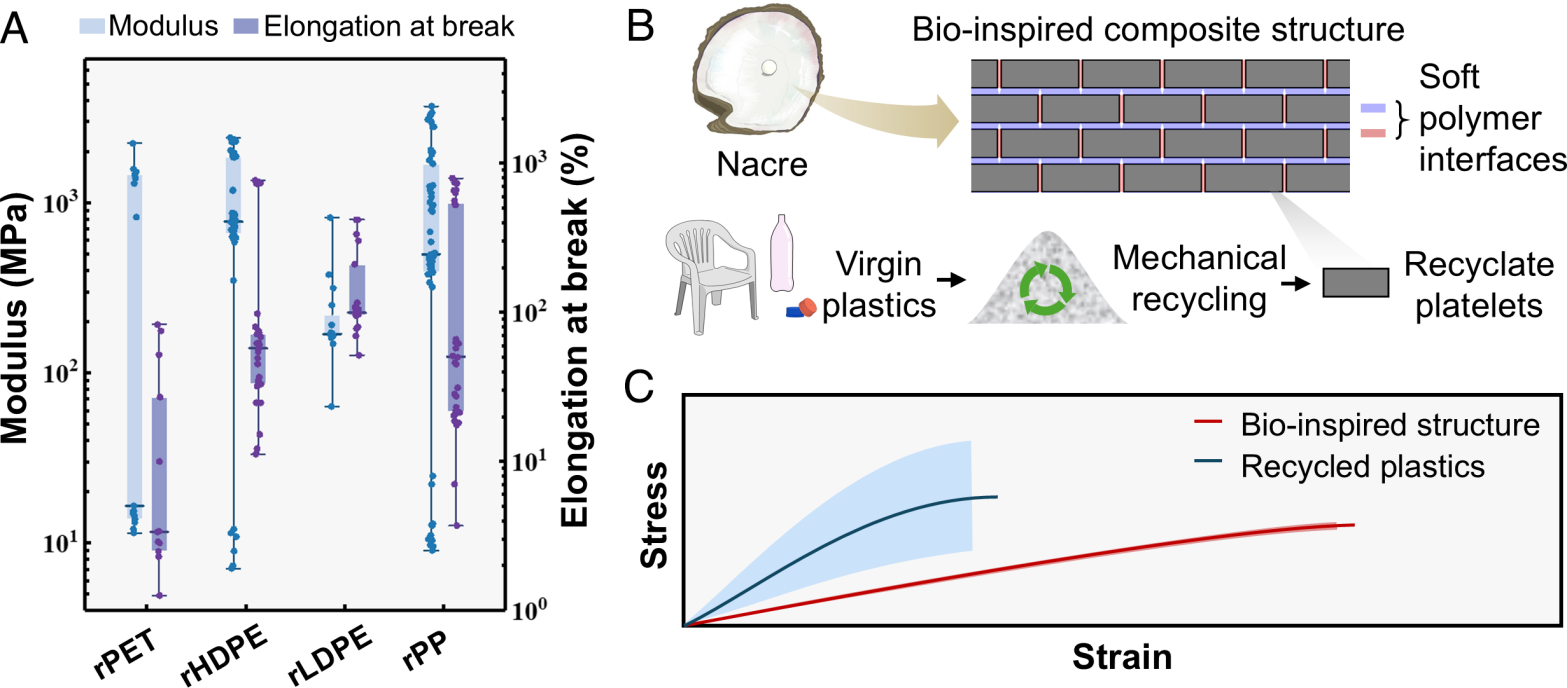
Suppressing variability through a bioinspired composite structure. (*A*) Summary of literature data (6–8, 37–44) demonstrating the significant variability in the mechanical properties of recycled plastics (details in *SI Appendix*, Fig. S1). Plot of the modulus and elongation at break of rPET, rHDPE, rLDPE, and recycled polypropylene (rPP). The mechanical property ranges shown include the cumulative effect of the variations due to origin, impurities, additive content, environmental exposure, and processing and experimental conditions of the tested materials reported in the studies. (*B*) Schematic of a nacre-inspired recyclable composite structure. Blocks of recycled plastics with uncertainty in their mechanical behavior are represented as hard bricks and are bonded by a softer polymer. (*C*) Conceptual schematic illustrating the broader uncertainty in the mechanical properties of recyclates compared to the narrower uncertainty that is desired in industrial applications. Solid lines and shaded bands represent mean values and SD, respectively.

Inspired by nacre’s hierarchical, brick-and-mortar structure, here, we introduce a universally applicable composite structure design for thermoplastics that dramatically reduces recyclates’ variability and delivers more uniform mechanical properties. In this scheme, recycled polymers form stiff platelets (bricks), while a thin layer of virgin soft polymers serve as the compliant phase (mortar) accommodating most of the deformation. Under load, these soft interfaces transfer stress between platelets, suppressing variability (35). To predict the effective modulus and strength of such bioinspired structures, as well as their variability, we developed an uncertainty-aware tension-shear-chain (UATSC) model that couples Monte Carlo simulations with variability data for brick modulus (sourced from an extensive literature survey; Fig. 1*A*) and a conservative 15% interfacial maximum traction and critical decohesion coefficient of variation (36). This framework enables parametric studies of geometric, interfacial, and material properties on composite effective modulus, strength, and variability suppression. We validated the UATSC predictions by fabricating and testing nacre-inspired composites using rHDPE platelets and polydimethylsiloxane (PDMS) mortar (Fig. 1*B*). Tensile and adhesion tests were used to calibrate model parameters, and the resulting experimental variability matched model forecasts. As a practical demonstration, we applied this brick-and-mortar design to industrial stretch film: the rHDPE brick-and-mortar structure achieved the same average modulus as the virgin HDPE stretch film, while its variability was reduced by 93% compared to its rHDPE constituents. This case study showcases our design’s ability to transform recycled materials of stochastic properties into products of robust quality (Fig. 1*C*). The suggested variability suppression approach, which is introduced here in the context of recycled plastics, cannot only facilitate virgin plastic substitution but also support design for recycling strategies and enable reliable performance modeling in circular product systems. An overview of the employed workflow is presented in Fig. 1.

## Results

### UATSC Network Model.

Nacre-inspired architectures have been widely investigated in composite structures to enhance overall strength, toughness, and impact resistance (45–48). These performance enhancements primarily arise from the synergistic interaction between the stiff platelets and the compliant interfaces. Accordingly, researchers have explored how changes in platelets’ geometry or interfacial strength influence performance (33, 49–53). However, nearly all of the nacre-inspired models assume the platelets and interfaces possess fixed, deterministic properties, ignoring the inherent stochastic variability that can be found in natural materials. Only a handful of studies have investigated the probabilistic aspects of brick-and-mortar architectures. Yet, these studies focus on varying the interfacial maximum traction to assess its influence on structural response (50), and failure behavior (33, 49). In addition, none of the prior studies investigated variability reduction in the context of recycled polymers. Consequently, a critical gap remains: Existing frameworks do not address how to design nacre-inspired structures that are robust to variations in constituent stiffness, adhesion, or geometry, leaving unexplored the challenge of achieving reliable performance under realistic, variable material conditions, such as recycled plastics (see a detailed comparison with prior nacre-inspired architectures and models in *SI Appendix*, Table S1).

To address this gap, we have developed a UATSC network model able to capture the interplay of deformable platelets of stochastic modulus and multiple stochastic interfacial parameters (maximum traction, elastic and critical separation limits) in brick-and-mortar architectures. Our approach builds upon a discrete element model, similar to ref. 33, that incorporates three types of elements, i.e., the hard elements (platelets), the cohesive tensile elements (CT), and the cohesive shear elements (CS).

**Fig. 2.**
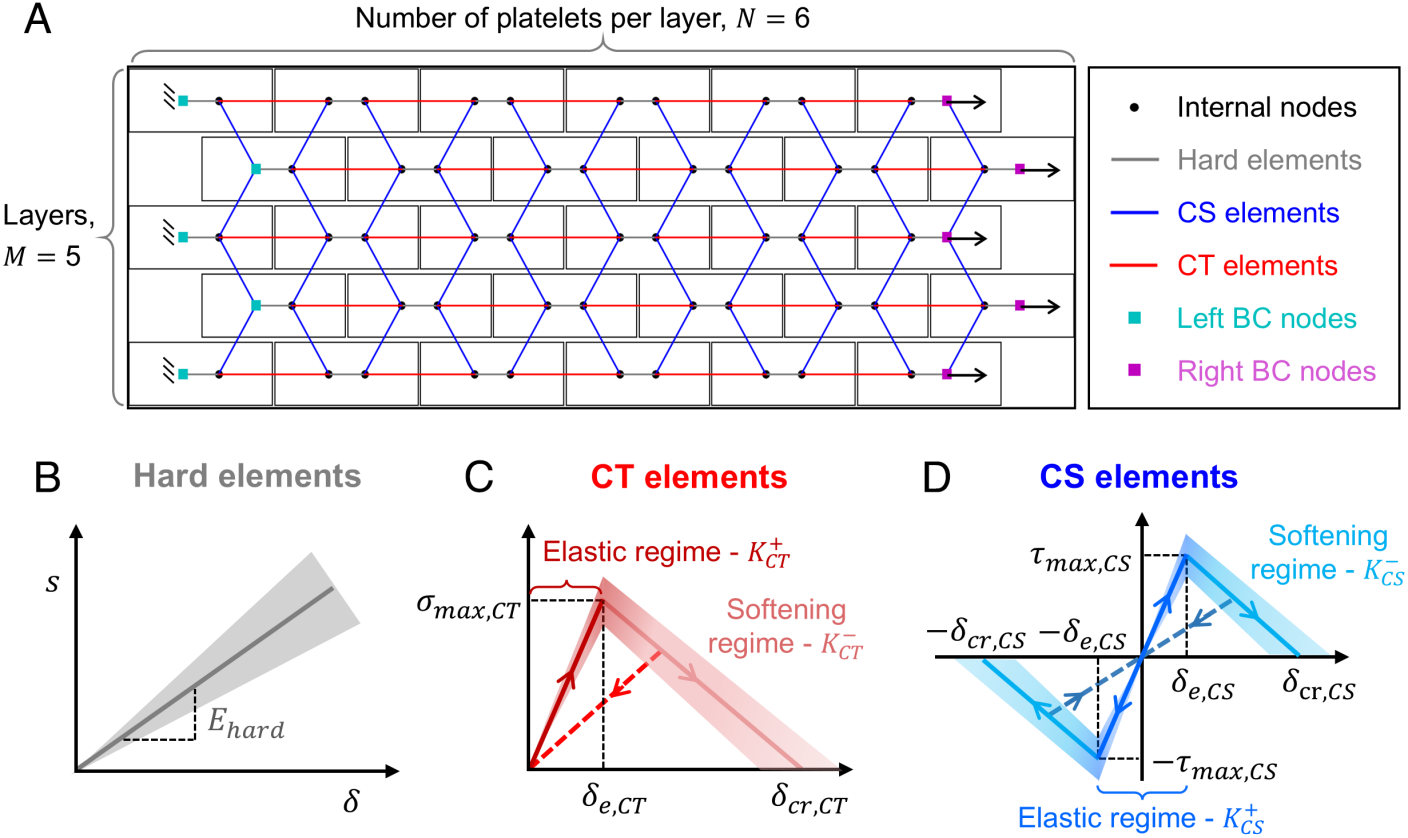
Components of the discrete network. (*A*) Connectivity of a staggered {6,5} network, comprising six platelets (hard elements) per layer and five layers, where N denotes the number of platelets per layer and M denotes the number of layers. Hard elements, cohesive tensile (CT) elements, and cohesive shear (CS) elements are represented by gray lines, red lines, and blue lines, respectively. Boundary nodes are highlighted: teal for fixed nodes and purple for nodes with externally applied displacement; BC stands for boundary conditions. (*B*) Stress–strain curve for uniaxial tension of the hard elements, where $E_{\mathrm{hard}}$ denotes the Young’s modulus. (*C*) Constitutive cohesive law of the CT elements featuring a linear regime, controlled by the maximum traction $\sigma_{max,CT}$, and the elastic separation $\delta_{e,CT}$ which define the stiffness $K_{\mathrm{CT}}^{+}$, and a softening regime defined by the reduced stiffness $K_{\mathrm{CT}}^{-}$ and the critical separation $\delta_{cr,CT}$. (*D*) Constitutive cohesive law of the CS elements featuring a linear regime, controlled by the maximum traction $\tau_{max,CS}$, and the elastic separation $\delta_{e,CS}$ which define the stiffness $K_{\mathrm{CS}}^{+}$, and a softening regime defined by the reduced stiffness $K_{\mathrm{CS}}^{-}$ and the critical separation $\delta_{cr,CS}$. Solid lines and shaded bands represent the mean values and SD, respectively, highlighting the variability in the properties of the recyclates, while dashed lines indicate the unloading path of the material.

The model represents a staggered network of hard elements with equal overlap (Figs. 1*B* and 2*A*). In each layer, the hard elements are lined up, side-by-side, and the next layer above is shifted creating the same amount of overlap on both sides of each hard element. The network is characterized by the number of hard elements in each layer, N, and the number of layers, M. An {N,M} network comprises N×M hard elements. CT and CS elements represent the compliant interfaces among the hard elements. CT elements connect hard elements within the same layer (horizontal direction), accounting for deformation across the same layer, while CS elements link hard elements between different layers (vertical or diagonal direction), capturing shear between the staggered layers of the network (Fig. 2*A*). The thickness of the cohesive elements is denoted as t. The total number of CT and CS elements in an {N,M} network is $N_{\mathrm{CT}}=\left(N-1\right)M$ and $N_{\mathrm{CS}}=\left(2N-1\right)\left(M-1\right)$, respectively. The CT and CS elements are characterized by bilinear cohesive constitutive laws defined by three parameters: a) the maximum traction, $\sigma_{max,CT}$ and $\tau_{max,CS}$, which represent the peak stresses the interface can sustain before damage initiation; b) the elastic separation, $\delta_{e,CT}$ and $\delta_{e,CS}$, where traction reaches its maximum during elastic loading, and the interface is in the onset of damage; and, c) the critical separation, $\delta_{cr,CT}$ and $\delta_{cr,CS}$, signaling full decohesion of the interface.

Hard elements are assumed to be linear elastic, characterized by a single material parameter, the Young’s modulus, $E_{\mathrm{hard}}$, while their contraction in the vertical direction, during uniaxial loading in the longitudinal direction, is considered negligible. The compliant CT and CS elements accommodate the bulk of the deformation, while the hard elements sustain the load without yielding

The $\left\{N,M\right\}$ network is modeled as a system of two-node elements, with each element resembling a generally nonlinear spring. Only horizontal displacements are considered under uniaxial tension. The system features $N_{\mathrm{DOF}}=2\left(N\times M\right)$ degrees of freedom (DoFs), corresponding to two horizontal displacement components per hard element (Fig. 2*A*). From the nodal displacements, the displacement jumps across the elements are calculated as, $\delta =u_{i}-u_{j}\forall i\neq j\epsilon \left[1,N_{\mathrm{DOF}}\right]$. The stress state and damage condition of each element are determined from the displacement jumps using a known constitutive relationship between deformation and the corresponding stress, as defined by Eqs. **1**–**4**. For the hard elements, 1D line elements are employed (Eq. **1** and Fig. 2*B*), and their performance is compared, across various displacement field states, to the stress predicted by the shear-lag model (Eq. **2**) (54), where the contributions of neighboring CT and CS elements determine the average stress in a hard element. The results demonstrate accurate predictions for elements in the inner layers of the structure, while the shear-lag model estimates slightly higher stresses in the top and bottom boundary layers. The stresses in the shear and tensile interfacial elements are determined directly from the traction-separation law, based on whether the element is intact, damaged, or fully destroyed (Eqs. **3** and **4**):

[1] $$\sigma_{\mathrm{Hard}}=\frac{E_{\mathrm{hard}}A}{l}\delta ,$$

[2] $$\sigma_{\mathrm{lag}}=\sigma_{\mathrm{CT}}+\frac{l}{h}\tau_{\mathrm{CS}},$$

[3] $$\begin{array}{c} \sigma_{\mathrm{CT}}=\left\{\begin{array}{c} \begin{array}{c} K_{\mathrm{CT}}^{+}\delta ,\;\delta \leq \delta_{e,CT}\\ K_{\mathrm{CT}}^{-}\delta ,\;\delta_{e,CT}<\delta <\delta_{cr,CT} \end{array}\\ 0\;\delta \geq \delta_{cr,CT} \end{array}\right., \end{array}$$

[4] $$\begin{array}{c} \tau_{\mathrm{CS}}=\left\{\begin{array}{c} \begin{array}{c} K_{\mathrm{CS}}^{+}\delta ,\;\delta \leq \delta_{e,CS}\\ K_{\mathrm{CS}}^{-}\delta ,\;\delta_{e,CS}<\delta <\delta_{cr,CS} \end{array}\\ 0,\;\delta \geq \delta_{cr,CS} \end{array}\right., \end{array}$$

where l is the platelet length, h is the platelet height, and A is the cross-sectional area of the platelet. For the interfacial elements, we employ a variable stiffness approach that captures the bilinear nature of the cohesive law (Fig. 2 *C* and *D*). The stiffness of an element during elastic deformation, denoted as $K^{+}$, is defined as the ratio of $\sigma_{max,CT},\tau_{max,CS}$ to $\delta_{e,CT},\delta_{e,CS}$, for CT and CS elements, respectively (Eqs. **5** and **6**). To model damage, we assume that the stiffness of the interfacial elements decreases as damage progresses; therefore, the element showcases softening. This reduced stiffness, $K^{-}$, of each element depends on the current displacement jump of the element and is calculated as the slope of the line connecting the origin to the point [s,δ] on the traction-separation curve (Eqs. **7** and **8** and Fig. 2 *C* and *D*).

[5] $$K_{\mathrm{CT}}^{+}=\frac{\sigma_{CT}}{\delta_{e,CT}},$$

[6] $$K_{\mathrm{CS}}^{+}=\frac{\tau_{\mathrm{CS}}}{\delta_{e,CS}},$$

[7] $$K_{\mathrm{CT}}^{-}=\frac{\delta_{cr,CT}}{\delta }\frac{\delta_{cr,CT}-\delta }{\delta_{cr,CT}-\delta_{e,CT}}K_{\mathrm{CT}}^{+},$$

[8] $$K_{\mathrm{CS}}^{-}=\frac{\delta_{cr,CS}}{\delta }\frac{\delta_{cr,CS}-\delta }{\delta_{cr,CS}-\delta_{e,CS}}K_{\mathrm{CS}}^{+}.$$

During loading, when strain localization occurs at certain interfacial elements, other elements can experience unloading. For damaged elements that undergo unloading, the stress corresponding to the displacement jump deviates from the bilinear traction-separation law and is instead determined by the unloading stiffness. The value of the unloading stiffness is defined based on the displacement jump, δ prior to unloading, considering negligent residual displacement (dashed line in Fig. 2 *C* and *D*).

To solve the system, we employ an iterative Newton scheme to enforce force equilibrium at each node. The equilibrium condition at each node is realized by iteratively updating the nodal displacements until the residual forces converge within a predefined tolerance. This approach is feasible due to the uniaxial tensile loading of the model and the underlying assumptions, which enable a streamlined mapping of stresses to forces, while considering only horizontal components in the equilibrium equations. The nodal displacements at each iteration are updated according to Eq. **9**:

[9] $$u_{\mathrm{new}}=u_{\mathrm{old}}-{\left[\nabla R\right]}^{-1}R,$$

where R is the residual force vector, and ∇R is the Jacobian matrix. The iterations continue until the second norm of the residual vector, ${\left|\left|R\right|\right|}_{2}$, is less than ε_R;_ = 10^−6^, ensuring convergence. The residual vector, R, represents the imbalance of forces at each node and is defined in Eq. **10**, where ***F*_*ext*_** is the vector of externally applied forces, and $F_{\mathrm{int}}$ is the vector of internal forces arising from the element stresses.

[10] $$R=F_{ext}-F_{int}.$$

The internal forces are computed by summing the contributions from all connected elements at each node, based on the stresses and orientations of the elements. For the uniaxial tension setup, R contains only horizontal force components. The internal forces are derived from the stress states of the CT, CS, and hard elements. These stresses are calculated based on the current δ using the traction-separation laws for the CT and CS elements and the linear constitutive model for the hard elements (Eqs. **1**, **3**, and **4** and Fig. 2 *B*–*D*). Through ∇R, the nonlinear relationship between the forces and the displacements, incorporating the stiffness reduction of damaged elements is accounted for.

Boundary conditions (BCs) are imposed by fixing the horizontal displacements of nodes along the left boundary and applying a specified displacement load, Δ, to nodes on the right boundary (Fig. 2*A*). The displacement load is incrementally increased in small steps until the predefined total applied elongation Δ is reached. This incremental loading ensures stability during the simulation and allows the system to accurately capture the nonlinear deformation and damage evolution in the network. The algorithm outputs the displacement values for all DoFs at each displacement increment, along with the element stresses, damage states (intact, damaged, unloaded, or destroyed), and the reaction force probed at the right boundary. These values are utilized to compute the structure’s effective modulus, elongation at break, and other metrics, such as yield stress and strength.

### Platelet and Interfacial Mechanical Property Variability.

Three sources of variability were assumed: 1) variability of $E_{\mathrm{hard}}$ of the hard elements, 2) variability of interfacial $\sigma_{max,CT}\;\mathrm{and}\;t_{max,CS}$, and 3) variability of interfacial $\delta_{cr,CT}$ and $\delta_{cr,CS}$.

Variability of $E_{\mathrm{hard}},$ is introduced (shaded bands in Fig. 2*B*) by drawing inspiration from the stochastic approaches of Luo and Bažant (49) and Yan et al. (33). As a first approximation, we sample $E_{\mathrm{hard}}$ using a two-parameter normal probability distribution, $N\left(\mu ,\sigma^{2}\right)$, which is parameterized based on literature data (6–8, 37–44). Although recycled polymers’ modulus variability may not be strictly normal, we decided to use such a distribution based on the central limit theorem (36): since many independent factors (e.g., processing conditions, recycling cycles, material lifetime, compositional fluctuations, etc.) contribute to the variation of $E_{\mathrm{hard}},$ their aggregate effect should approximate a normal distribution. Adopting a normal form also enables symmetric outlier handling and the use of standard statistical tools. Even if the true distribution deviates from normal, the qualitative insights and relative quantitative predictions remain accurate. The probability density function (PDF) of the normal distribution is given by Eq. **11**:

[11] $$P_{N}\left(x\right)=\frac{1}{\sqrt{2\pi \sigma^{2}}}e^{-\frac{{\left(x-\mu \right)}^{2}}{2\sigma^{2}}},$$

where x is the random variable, and μ,σ is the mean value and the SD of the population respectively.

We further consider that the inherent variability of the recyclates introduces uncertainty in $\sigma_{max,CT},t_{max,CS}$ and $\delta_{cr,CT},\delta_{cr,CS}$ (shaded bands in Fig. 2 *C* and *D*), as the interfacial behavior is closely tied to the properties of the substrate (55, 56). We sample $\delta_{cr,CT}$ and $\delta_{cr,CS}$ based on Eq. (**11**) and further assume that $\sigma_{max,CT}$ and $\delta_{max,CS}$ follow a Weibull distribution, W(m,s) (PDF shown in Eq. **12**), given its suitability for modeling fracture-related properties (33, 49, 57)

[12] $$P_{W}\left(x\right)=\frac{mx^{m-1}}{s^{m}}e^{-\frac{x^{m}}{s^{m}}},$$

where m,s are the shape and scale parameters of the distribution, respectively. The shape parameter, m, controls the distribution’s form—lower values yield heavier tails and larger relative variance, while higher values produce a more concentrated curve—and the scale parameter, s, stretches the distribution and influences the mean value.

To compute the effective properties of the network, we perform individual experiments of the network featuring randomized mechanical properties for each of the constituents. For every realization, the structure’s effective modulus, $E_{\mathrm{eff}}$ is computed as the slope of the linear region of the stress–strain curve (Eq. **13**).

[13] $$E_{\mathrm{eff}}=\frac{\sigma_{\mathrm{yield}}}{\Delta_{\mathrm{yield}}}L,$$

where $\sigma_{\mathrm{yield}}$ represents the stress at the last increment before damage is detected in the structure, here defined as the yield stress, and $\Delta_{\mathrm{yield}}$ is the corresponding displacement. The initial length of the structure, L, corresponds to the cumulative length of the hard elements and CT elements along a single layer, while considering the staggered arrangement of layers.

The strength, $\sigma_{\max}$, is defined as the maximum stress observed during the experiment and the elongation at break, $\varepsilon_{\max},$ is defined as the ratio of the change in the structure’s length to its original length, at the point of failure. The individual $E_{\mathrm{eff}},\varepsilon_{\max}$ and $\sigma_{\max}$ values are then aggregated across all realizations to compute the statistics: the mean value, E[∙] = μ, is calculated as the average of the individual values, while the variance of $V\left[∙\right]=\sigma^{2}$, is computed as the square of the SD of the samples indicating the spread about μ. We quantify the stochasticity of the network’s properties by the ratio of the coefficient of variation, CV, (Eq. **14**) of the material to that of the effective property ($E_{\mathrm{eff}},\varepsilon_{\max}$, $\sigma_{\max}$).

[14] $$CV=\frac{\sigma }{\mu }.$$

Assuming that these three sources of variability—hard elements’ $E_{\mathrm{hard}}$, cohesive elements $\sigma_{max,CT},t_{max,CS}$, and $\delta_{cr,CT},\delta_{cr,CS}$—are statistically independent, the total introduced variability in the response can be estimated through the root-sum-square of the individual $CV_{i}$ (Eq. **15**), where i indicates sources of variability in the network.

[15] $$CV_{\mathrm{total}}=\sqrt{\sum_{i}CV_{i}^{2}}.$$

By explicitly incorporating stochasticity at the component level, the developed UATSC model enables predictive understanding of how heterogeneity propagates to structural performance.

### Impact on Effective Modulus.

**Fig. 3.**
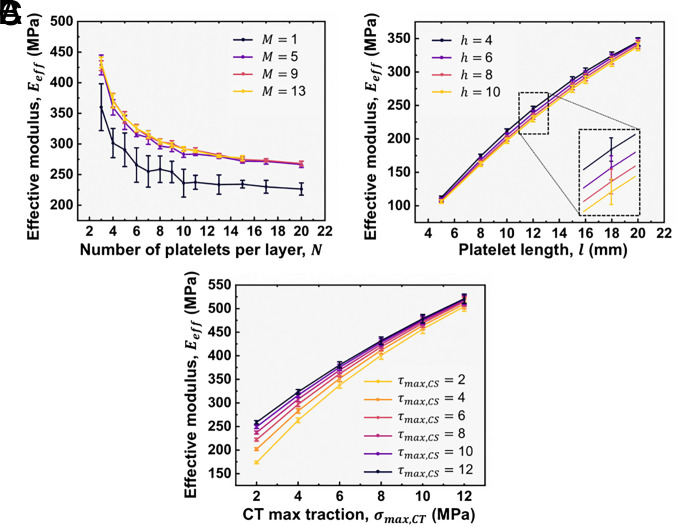
Sensitivity analysis of the effect of network’s parameters on the structure’s effective modulus, $E_{\mathrm{eff}}$. (*A*) Network size: variation in the effective modulus, $E_{\mathrm{eff}}$, with the number of platelets per layer, N, and the number of layers, M. The results highlight the convergence of structural properties and significant suppression of variability for larger network sizes. (*B*) Platelet dimensions: various combinations of platelet height, h, and length, l, are analyzed; h has a negligible effect on the mean value of $E_{\mathrm{eff}}$ while no parameters influence the variability levels. (*C*) Interfacial properties: $\sigma_{max,CT},\tau_{max,CS}$, influence $E_{\mathrm{eff}}$ by altering $K_{\mathrm{CT}}^{+}$ and $K_{\mathrm{CS}}^{+}$. Among these, increasing $\sigma_{max,CT}$ has the most pronounced effect, indicating that CT elements are the primary contributors to the structure’s $E_{\mathrm{eff}}$.

We tested the impact of the network’s parameters with the greatest expected influence on $E_{\mathrm{eff}}$, i.e., N,M, l, h, $\sigma_{max,CT},\tau_{max,CS},$ through a sensitivity analysis. The arrangement of the platelets is considered uniform and periodic. We divide these parameters into three categories: the network size (N,M—Fig. 3*A*), the platelet dimensions (l, h—Fig. 3*B*), and the interfacial properties ($\sigma_{max,CT},\tau_{max,CS},$—Fig. 3*C*). To efficiently perform a high number of simulations, we conducted a convergence analysis to determine the sample size required for achieving reliable results for the model’s variability, and selected 200 simulations, as the optimal sample size (*SI Appendix*, Fig. S2).

First, we varied the network size, while keeping all other parameters constant (*SI Appendix*, Table S2). Smaller networks exhibit higher variability, which can be explained by the transition from simple connectivity configurations, such as the serial chain and alternative column, to more complex ones that constrain variability dispersion (Fig. 3*A*; *Examination of UATSC Model Performance in Limiting Cases and Model Validation*). Additionally, increasing N reduces $E_{\mathrm{eff}}$ for a given M, as the added hard elements resemble the serial chain configuration where $E_{\mathrm{eff}}$ is lower than the $E_{\mathrm{hard}}$ and $K_{\mathrm{CT}}^{+}$ of individual constituents. In contrast, increasing M, beyond M≥5, for a given N has a minimal effect on $E_{\mathrm{eff}}$ due to the more complex connectivity of the elements. Overall, increasing the network size leads to convergent $E_{\mathrm{eff}}$.

Then, we examined the sensitivity of the network’s properties to the geometric parameters of the platelets (Fig. 3*B*), by varying h, and l, with the remaining properties as listed in *SI Appendix*, Table S2. A {10,5} network size is chosen for its balance between reduced variability and adequate $E_{\mathrm{eff}}$, while being suitable as a tensile specimen across various l. Increasing l leads to higher $E_{\mathrm{eff}}$, as the contribution of CS elements is influenced by the aspect ratio, ρ=l/h, of the platelets (54). However, while increasing h enhances $K_{\mathrm{CT}}^{+}$, it simultaneously increases A, minimizing its overall contribution to the structure’s $E_{\mathrm{eff}}$. This is verified by $E_{\mathrm{eff}}$ fluctuations which show a clear dependence on l, while remaining largely insensitive to variations in h. Variability in the $E_{\mathrm{eff}}$ does not increase, as the CV remains nearly constant across different platelet dimensions.

Finally, we examine the effect of $K_{\mathrm{CT}}^{+}$ and $K_{\mathrm{CS}}^{+}$ (Fig. 3*C*) by varying s in the distributions of $\sigma_{max,CT},\tau_{max,CS}$ while keeping $\delta_{e,CT}$ and $\delta_{e,CS}$ constant. The remaining properties are listed in *SI Appendix*, Table S2 and the {10,5} network size is maintained for consistency. The results show that $\sigma_{max,CT}$ has the greatest impact on $E_{\mathrm{eff}}$, as CT elements directly resist deformation during uniaxial tension. In contrast, $\tau_{max,CS}$ plays a secondary role due to CS element shear slip deformation. As $K_{\mathrm{CT}}^{+}$ increases, $E_{\mathrm{eff}}$ becomes dominated by the tensile behavior of the CT elements, with the CS elements being primarily responsible for stress redistribution rather than contributing critically to the $E_{\mathrm{eff}}$. This is supported by the observation that $E_{\mathrm{eff}}$ fluctuations are sensitive only to $\sigma_{max,CT}$, while the CV remains nearly constant.

### Impact on Structure’s Strength.

**Fig. 4.**
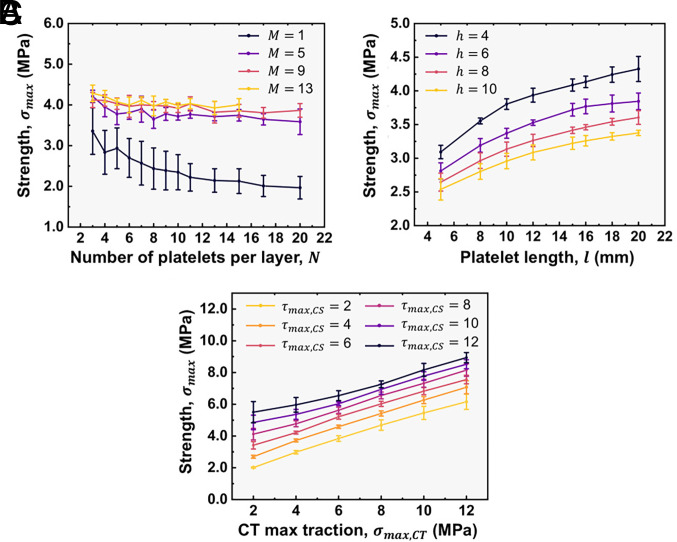
Sensitivity analysis of the effect of network’s parameters on the structures’ strength, $\sigma_{\max}$. (*A*) Network size: variation in $\sigma_{\max}$ with N and M. The results highlight the convergence of structural properties for larger network sizes with reduced variability for M≥5. (*B*) Platelet dimensions: various combinations of h, l, are analyzed; structures with lower h and higher l exhibit higher $\sigma_{\max}$ suggesting the aspect ratio ρ=l/h is a critical parameter. (*C*) Interfacial properties: both $\sigma_{max,CT},\tau_{max,CS}$ parameters, directly influence $\sigma_{\max}$. Notably, increasing $\tau_{max,CS}$ has the most pronounced effect, indicating that CS elements dominate the large-deformation regime dictating $\sigma_{\max}$.

To investigate the effect of network’s parameters on $\sigma_{\max}$ we perform a similar sensitivity analysis while also accounting for $\delta_{cr,CT},{\delta d}_{cr,CS}$ and their variability. We have grouped results based on network size (Fig. 4*A*), platelet dimensions (Fig. 4*B*), and interfacial properties (Fig. 4*C*). Each configuration is identical to the corresponding one used for the $E_{\mathrm{eff}}$ with the exception that in all instances $\delta_{cr,CT},$ and $\delta_{cr,CS}$ are now considered to be normally distributed N (0.50, $0.075^{2}$) featuring an elevated CV of 15%.

The variability in $\sigma_{\max}$ decreases with network size and, especially for configurations with M≥5, $\sigma_{\max}$ aligns more closely with the strongest link than the weakest link criterion (Fig. 4*A*) while featuring a convergent mean value. For smaller network sizes, oscillations of the mean value of $\sigma_{\max}$ can be attributed to element realizations whose properties’ values are situated at the lower end of their respective distributions thereby influencing heavily the response. M serves as the primary factor in suppressing variability by increasing the complexity of element connectivity, hence reducing the influence of weaker elements and inhibiting error dispersion. N also contributes to variability suppression, although to a lesser extent, by introducing additional load-bearing elements that help redistribute stress across the interfacial regions.

The influence of platelet dimensions on $\sigma_{\max}$ differs from their effect on the $E_{\mathrm{eff}}$. Notably, both h and l have a significant impact on $\sigma_{\max}$ (Fig. 4*B*). This relationship can be understood through ρ; higher values result in increased overlap between platelets and interfacial regions, ultimately enhancing load transfer and $\sigma_{\max}$. This trend is consistent with observations concerning nacre and nacre-inspired structures reported in the literature (30, 33, 48, 58). Although increasing h may enhance $\sigma_{max,CT}$, it is $\tau_{max,CS}$ that primarily governs $\sigma_{\max}$. CS elements benefit from a higher ρ therefore increasing h has an adverse effect on the $\sigma_{\max}$. In terms of variability reduction, hard elements’ dimensions do not influence the stochastic profile of the network, as they do not alter the connectivity or stress-sharing mechanisms among elements within the network.

Finally, $\sigma_{max,CT},\tau_{max,CS}$ directly influence $\sigma_{\max}$. The relative values of $\sigma_{max,CT}$ and $\tau_{max,CS}$ determine the dominant failure modes (Fig. 4*C*). When both parameters are similar, the CT elements primarily govern $E_{\mathrm{eff}}$ which is progressively reduced as they undergo damage, while the CS elements dominate during the postelastic regime dictating the $\sigma_{\max}$. This configuration is a preferred compromise between $\sigma_{\max},E_{\mathrm{eff}},$ manufacturability and variability reduction. Configurations with a high $\tau_{max,CS}$/$\sigma_{max,CT}$ ratio typically exhibit lower *E_eff_*, with shear deformation through the CS elements dominating the mechanical response. The resulting stress–strain curves are smoother, as CT element failures contribute minimally beyond the linear regime. In contrast, configurations with a low $\tau_{max,CS}$/$\sigma_{max,CT}$ ratio display a stiffer response but limited stretchability, as the CT elements fail early with little deformation, while the CS elements have already exceeded their stress threshold. In both instances, $\sigma_{\max}$ is compromised, as early element damage restricts effective load transfer.

### Model-Experiment Comparison.

As a proof-of-concept, we used commercially available rHDPE to fabricate brick-and-mortar structures, since rHDPE is among the most widely used polymer resins in the United States (59). The developed structures were characterized under uniaxial tension for the experimental validation of the UATSC model. The tensile properties and statistical variability of the rHDPE material were also characterized through uniaxial tensile testing, and the resulting data were used as direct input for $E_{\mathrm{eff}}$ in the modeling framework.

To fabricate the rHDPE brick-and-mortar structures, rHDPE sheets were cut into platelet-sized components. For the interfacial layer, we used PDMS, a soft commercial elastomer known for its stretchability and adhesion properties. To assess the suitability of PDMS as an adhesive for rHDPE, we performed lap-shear tests to quantify the interfacial traction-separation behavior between the two materials. These measurements provided the distribution of $\sigma_{max,CT},\tau_{max,CS}$ and $\delta_{cr,CT},\delta_{cr,CS}$ as well as the values of $\delta_{e,CT}$ and $\delta_{e,CS}$ needed to calibrate the CT and CS elements in the model (*Characterization of Platelet and Interface Properties*). The resulting composite structures were fabricated by embedding aligned rHDPE platelets within a PDMS matrix, following the architectural guidelines derived from the simulation-based design in a {8,5} network. The dimensions of the fabricated structure and the corresponding parameters of the model are summarized in *SI Appendix*, Table S3. The resulting volume ratio, $V_{\mathrm{plat}}/V_{\mathrm{total}}$, defined as the ratio of the platelets’ volume to the total structure’s volume is 70.7%. The mechanical behavior of the developed structure was then evaluated under uniaxial tensile loading to enable a direct comparison with model predictions (*Fabrication and Testing of Bio-Inspired Composite Structure*).

**Fig. 5.**
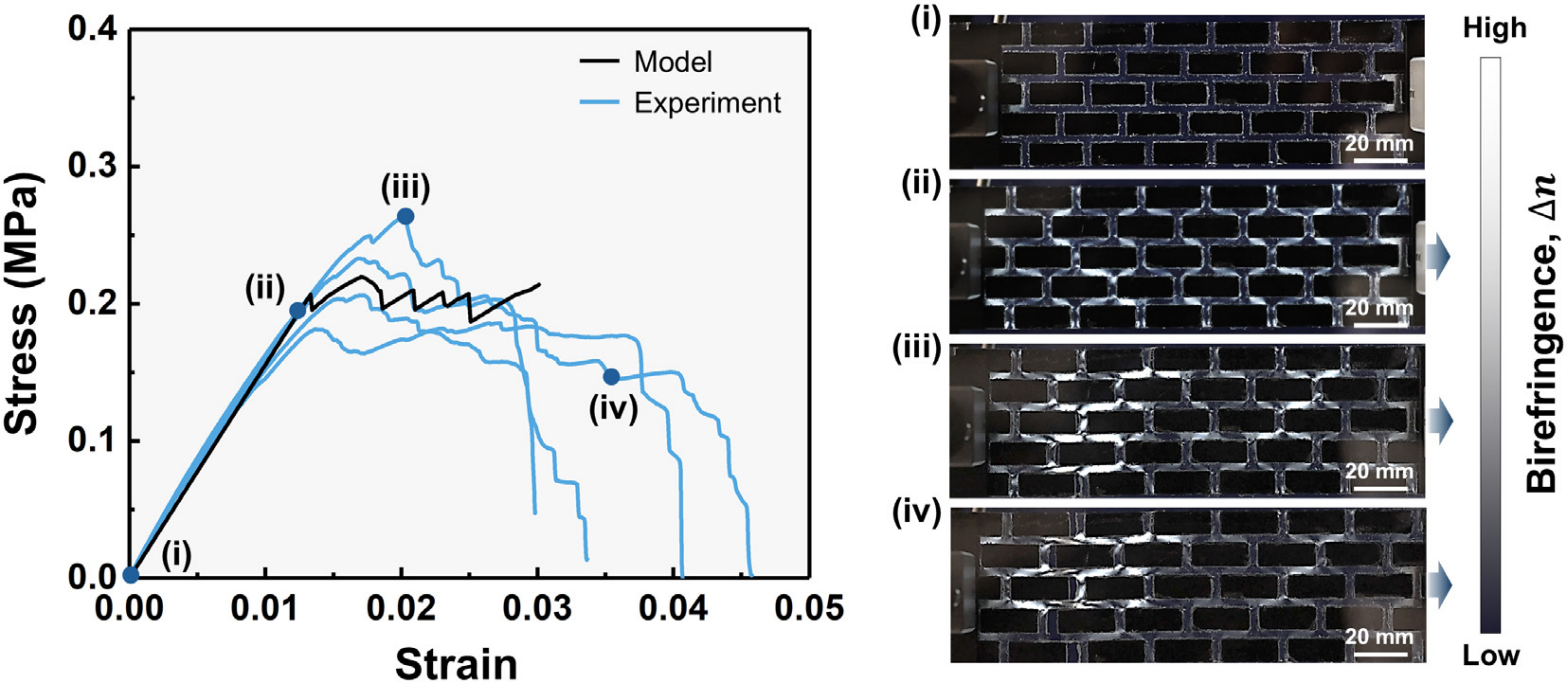
Comparison between experimental tensile tests and UATSC model predictions for the bioinspired composite structure. The model captures the average stress–strain response of the bioinspired structure and closely follows the onset and initial damage observed experimentally. Snapshots of the physical specimen at key stages of deformation highlight the evolution of damage, which agrees with the simulated behavior (*Insets i*–*iv*). Initially the structure is unstressed (*Inset i*) with no apparent residual birefringence. The response consists of a linear region where elements are almost equally loaded (*Inset ii*), an initial damage regime due to decohesion of CT elements (*Inset iii*) and finally localized yielding (*Inset iv*) which leads to fracture. The brightness level at the interfaces indicates the birefringent effect which can be qualitatively associated with developed stresses, i.e., brighter regions have higher stress. The coefficient of variation, CV, of $E_{\mathrm{eff}}$ (0.005 strain point) is reduced to 4% compared to the initial variability in the platelets (CV = 18%) verifying both the applicability of the model and the structure’s potential to suppress such variability. A representative experiment demonstrating the real-time deformation sequence is provided in Movie S1.

The experimental results closely followed the model predictions in the small strain region with reasonable deviations in the damage regime. The mean value of the experimentally observed $E_{\mathrm{eff}}$ matched the corresponding simulated mean value within an 8% deficit, validating the model’s ability to capture the network’s elastic behavior (Fig. 5). Photoelasticity was employed during testing to qualitatively assess the loading intensity in real time (the birefringence, Δn, was monitored). The *Insets* in Fig. 5 show photoelastic images at key strain stages, which highlight the evolution of loading paths and damage localization that correspond to distinct phases of the simulated response (*SI Appendix*, Fig. S3). The stress–strain curves from the physical specimens reproduced the deformation patterns predicted by the UATSC model with the pattern of loaded CS and CT elements in the linear region (*Inset ii*, Fig. 5) followed by breaking of the CT elements (*Inset iii*, Fig. 5), then localized yielding (*Inset iv*, Fig. 5) and fracture. A representative experiment demonstrating this sequence is provided in Movie S1. The CV of the fabricated specimens’ $E_{\mathrm{eff}}$ (0.005 strain point), was reduced to 4%, compared to the original 18% in the rHDPE feedstock. This suppression of variability is consistent with the simulation results, which predicted that the selected {8,5} network would feature a CV of 3% for $E_{\mathrm{eff}}$. In terms of the experimental $\sigma_{\max}$, the mean value μ= 0.22 MPa although close to the mean $\sigma_{max,CT},\tau_{max,CS}$, is 2 times higher than that of the weakest element while also featuring significant variability reduction (CV= 13%) despite the introduction of uncertainty both during fabrication and due to the sample size. Notably the model’s prediction for $\sigma_{\max}$, featuring a CV of 11%, lies within the observed experimental values. Finally, the model overestimates the response at excessive damage, i.e., when the CT elements have failed and CS elements exhibit localized softening. This is reasonable given the small-strain kinematic assumptions, which neglect changes in the deformation gradient at large strains – effects that, if included, would more accurately capture the failure of the structure. Overall, these results confirm the model’s agreement with experiments and demonstrate its potential as a design tool for engineering uncertainty-aware recycled structures.

### Case Study: Applying the UATSC Model to Industrial HDPE Stretch Film.

Industrial stretch film is a thin, stretchable sheet, primarily composed of polyethylene (HDPE, LDPE, and LLDPE), and widely used to stabilize and protect goods during packaging and shipping (60). In the United States alone, annual production exceeds 4 million tons (61), corresponding to a market value of $4.47 billion (62). Although polyethylene stretch film is technically recyclable, efforts to recycle it face major setbacks, as contamination, degradation, and property inconsistency in postconsumer films limit its mechanical performance (63).

Throughout its lifecycle, environmental stressors, such as prolonged ultraviolet (UV) exposure, induce structural changes in polymer stretch-films. UV aging and photo-oxidation increase crystallinity and crosslink density, driving the modulus higher while simultaneously reducing ductility (64, 65). Subsequent recycling exacerbates these issues (66, 67). As an example, rHDPE modulus varies substantially with its degradation history (Fig. 1*A*). Moderate aged rHDPE, exhibits a modulus 110 to 140% of the virgin value (65, 68, 69), whereas highly crystalline or UV-aged samples can reach up to 180% (64). At the same time, $\varepsilon_{\max}$ drops to below 0.1 (64, 65, 68, 69). Such unpredictable change in mechanical performance renders direct use of rHDPE for stretch film impractical, so waste film is often downcycled into thicker-gauge commercial films or trash bags. Therefore, we applied our framework to assess whether rHDPE can be used in industrial stretch films, to prevent its downcycling, aiming at variability suppression and the ability to meet select performance targets related to virgin HDPE films.

Our primary target was to reduce mechanical property variability, setting a CV of 5% for $E_{\mathrm{eff}}$ which corresponds to a 70% reduction with respect to the platelets’ $E_{\mathrm{hard}}$. In parallel, we defined a set of performance targets to assess the feasibility of meeting stretch film requirements. Specifically, we targeted an $E_{\mathrm{eff}}$ of ~550 MPa as a conservative goal given that commercial film moduli typically span 400 to 700 MPa (64, 70, 71) and an $\varepsilon_{\max}$ of ~100% consistent with values reported for HDPE stretch films (64). While these targets focus on specific, convenient property metrics, they do not fully capture the comprehensive performance requirements of stretch films, nor do they necessarily reflect the full property profile of virgin HDPE.

**Fig. 6.**
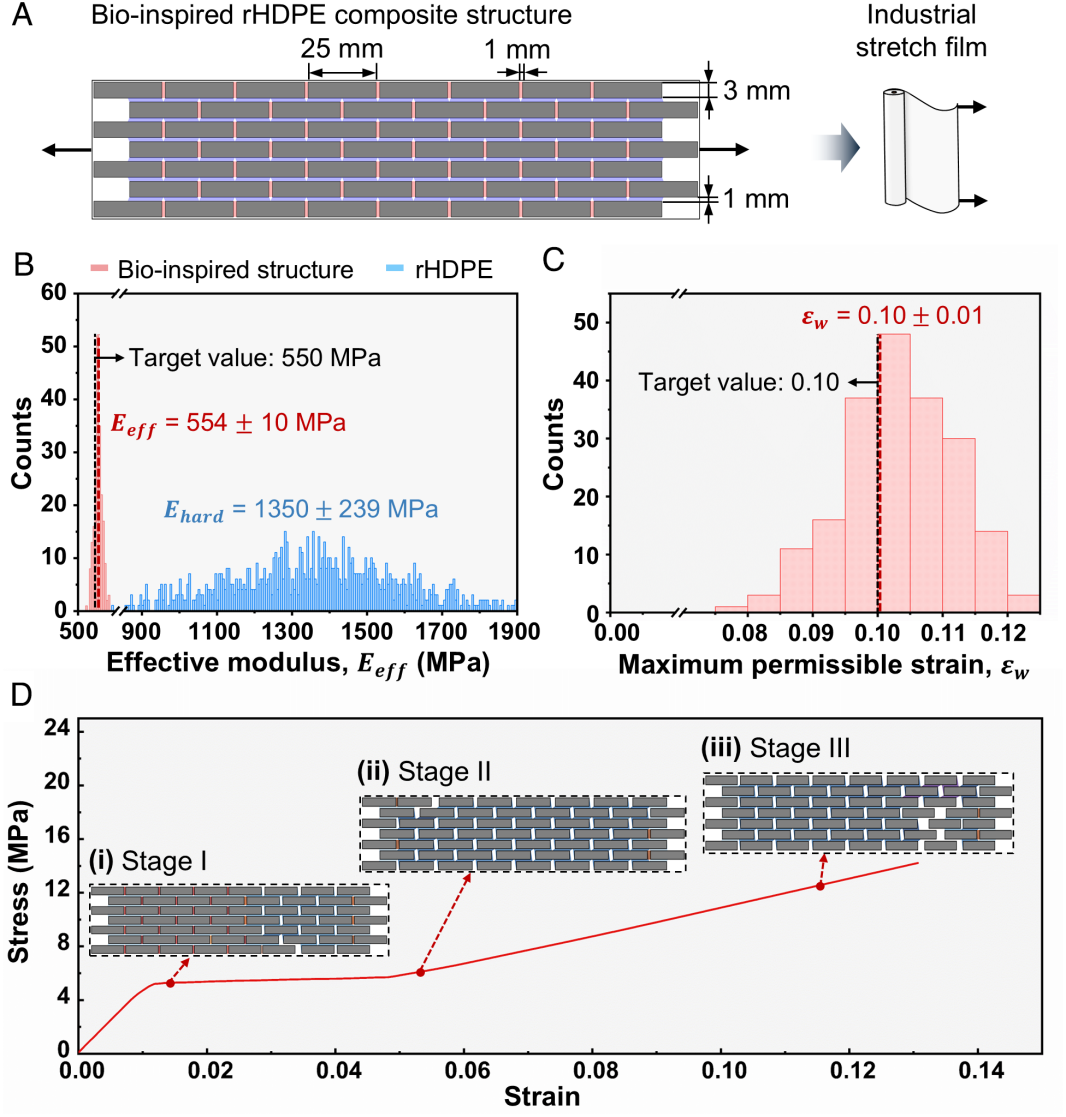
Reliable mechanical performance of bioinspired rHDPE composite for stretch film applications. (*A*) The bioinspired composite structure, utilizing rHDPE as hard platelets. (*B*) Distribution of $E_{\mathrm{eff}}$ of the bioinspired structure (red) for 200 network simulations and $E_{\mathrm{hard}}$ of the rHDPE (blue). The target modulus of the (virgin) HDPE stretch film is 550 MPa (black dashed line) while the mean value of the composite is 554 MPa (red dashed line). The CV of $E_{\mathrm{eff}}$ is reduced to approximately 2%, which corresponds to the typical range of properties during testing of standard materials. (*C*) Histogram of the maximum permissible strain $\varepsilon_{W}$ of the bioinspired structure for 200 network simulations. The target $\varepsilon_{W}$ of 0.10 is met by the bioinspired rHDPE composite. (*D*) Averaged stress–strain curve of the bioinspired structure up to the point of severe damage and localized yielding. The response features three stages: stage I shows linear elastic behavior dominated by CT elements (*Inset i*); in stage II, progressive CT elements’ failure leads to a stress plateau, with load redistributed to CS elements operating in their elastic region (*Inset ii*); stage III involves CS elements damage (purple in *Inset iii*), strain localization, and eventual failure.

Although $\varepsilon_{\max}$ is of primary interest for stretch films, fluctuations in $E_{\mathrm{eff}}$ directly influence its mean value by promoting uneven deformation and early failure (*SI Appendix*, Fig. S4). Hence controlling the distribution of $E_{\mathrm{eff}}$ is important as well. We also introduce the maximum permissible strain, $\varepsilon_{W}$. Here, $\varepsilon_{W}$ represents the strain threshold prior to the onset of excessive damage and marks the upper limit of the model’s predictive accuracy. This is because progressive damage and failure in some CS elements compromises the stress-distribution mechanism of the structure. The stress in the hard elements increases and, due to the relatively strong interfaces used, approaches the yield stress of the platelets, beyond which the resulting plastic deformation is not captured. Therefore, $\varepsilon_{W}$ serves as a conservative threshold beyond which the onset of large deformations challenges the validity of the model’s assumptions, while a confident estimate of $\varepsilon_{\max}$ remains unattainable within the current modeling framework. Accounting for the model’s limited predictive capability of $\varepsilon_{\max}$, we aimed to achieve an $\varepsilon_{W}\geq 0.1$ to match the $\varepsilon_{\max}$ of environmentally degraded HDPE films (64) To satisfy the set requirements, we implemented an {8,7} network for rHDPE platelets bonded by compliant polymeric interfaces (Fig. 6*A*). Sensitivity analyses (Figs. 3 and 4), show that this configuration converges toward a stable, reproducible mechanical response. We modeled the distribution of $E_{\mathrm{hard}}$ using experimentally measured properties of our rHDPE material, while the mean values of $\sigma_{max,CT},\tau_{max,CS},\delta_{cr,CT},\delta_{cr,CS}$ were tuned so that the composite’s predicted stress–strain curve matched the target behavior (*SI Appendix*, Table S4). To capture variability arising from the recycling process and possible fabrication defects, we introduced stochasticity by drawing $\sigma_{max,CT},\tau_{max,CS}$ from a Weibull distribution and assigning $\delta_{cr,CT},\delta_{cr,CS}$ a normal distribution with a CV of 10%. Under these assumptions, the overall network-level variability introduced is approximately 26% (Eq. **15**). The full set of model parameters appears in *SI Appendix*, Table S4 and the structure features a $V_{\mathrm{plat}}/V_{\mathrm{total}}=$ 82.4%.

We conducted 200 simulations to obtain $E_{\mathrm{eff}}$, $\varepsilon_{W}$, and their variability. The $E_{\mathrm{eff}}$ of the composite structure is 554 ± 10 MPa, and the $\varepsilon_{W}$ is 0.10 ± 0.01. $E_{\mathrm{eff}}$ features a CV of 2%, which is well within the anticipated specimen-to-specimen variability of any material and demonstrates 93% less variability compared to the total introduced variability while matching the modulus of the virgin HDPE film (Fig. 6*B*). Moreover, $\varepsilon_{W}$ exhibits substantially reduced variability, with a CV ratio of 0.32 relative to the total introduced variability, corresponding to about 68% variability reduction $\varepsilon_{W}$. While the proposed design achieves robust mechanical behavior with significantly suppressed variability, and even though $\varepsilon_{W}$ is conservative, the results do not fully reproduce the $\varepsilon_{\max}$ observed in virgin HDPE films. Still these findings constitute an improvement with respect to the $\varepsilon_{\max}$ of environmentally degraded HDPE films (64). With ideally tuned interfacial properties, full 3D modeling, and manufacturing tailored to film-grade standards, we expect the attainable $\varepsilon_{\max}$ to improve significantly.

The three-stage stress–strain curve of the brick-and-mortar structure is shown in Fig. 6*D*. During stage I, the composite structure behaves in a linear elastic manner, with the CT elements primarily bearing the load (*Inset i*). During stage II, the CT elements (red elements in Fig. 6*D*) reach their traction threshold, $\sigma_{max,CT}$, initiating gradual damage that propagates through the structure. This results in a plateau in the stress–strain response, persisting until all CT elements have failed. During this phase, load is progressively redistributed from the damaged CT elements to the still-elastic CS elements, enabling continued stretching of the composite (transition from *Inset i* to *Inset ii*). Notably, the failure of CT elements neither impairs structural functionality nor compromises load-bearing capacity. During stage III, some CS elements begin to experience damage (purple elements in *Inset iii*), leading to strain localization and the onset of nonlinear deformation. As this stage progresses, far-field elements may begin to unload, introducing complex stress states. The localized yielding concentrates stress within affected regions, serving as a precursor to eventual structural failure. This case study demonstrates—through careful parameter tuning to maintain performance standards at every stage of mechanical response—the potential of the developed framework to produce reliable stretch-film structures from recyclates exhibiting significant variability.

## Discussion

The UATSC model’s bioinspired, brick-and-mortar architecture suppresses variability in recycled materials by exploiting the interconnected network of CT and CS elements. In this scheme, CT elements dictate $E_{\mathrm{eff}}$ and elastic response, while CS elements redistribute stress and accommodate deformation as damage progresses. Together, they enable controlled failure modes that reduce performance scatter.

Two key design parameter groups are $K_{\mathrm{CT}}^{+},K_{\mathrm{CS}}^{+}$ and $\sigma_{max,CT},$$\tau_{max,CS}$. Because adhesion to recycled plastics depends on substrate chemistry, we recommend silicone-based adhesives, epoxies, or polyurethanes (72, 73) for hydrophobic polymers (e.g., HDPE, LDPE, PET) and hydrogel-based adhesives (74, 75) for hydrophilic polymers [e.g., polyvinyl alcohol (PVA), polyacrylic acid (PAA)]. Guided by UATSC simulations, $K_{\mathrm{CT}}^{+}$ should be about 1.5 to 2 times larger than $K_{\mathrm{CS}}^{+}$, yet still an order of magnitude lower than $E_{\mathrm{hard}}$ to ensure efficient load transfer. Moreover, $\sigma_{max,CT},\tau_{max,CS}$ must remain below roughly 30% of the yield (or peak) stress of the constituent polymer so that failure initiates at the interfaces, triggering load redistribution and variability suppression. These choices yield characteristic “peak-plateau-peak” instead of “two-peak” stress–strain curves (48). We therefore recommend characterizing interfacial performance via lap-shear testing (*Characterization of Platelet and Interface Properties*) and tuning adhesive selection based on UATSC predictions and the intended service conditions.

Beyond interfacial properties, platelet geometry also influences overall performance. Increasing l increases $K_{\mathrm{CS}}^{+}$ and consequently $E_{\mathrm{eff}}$ due to the larger CS-hard element overlap, while although increasing h increases $K_{\mathrm{CT}}^{+}$, it also increases A yielding negligible merits. Thus, tuning ρ by careful selection of both l,h can lead to optimal $E_{\mathrm{eff}}$ and $\sigma_{\max}$. Meanwhile, hard elements, modeled as linear elastic, can sustain stresses with minimal deformation. In applications involving large strains, such as stretch films, a finite-deformation constitutive model would be required to accurately capture the response of the structure at excessive damage. The current model is limited by a) its linear elastic constitutive model for the hard elements and b) its use of a small-strain kinematic framework, which neglects geometric effects at large deformations. While these limitations affect the accuracy of predictions in the excessive-damage regime, they do not, however, compromise the variability suppression mechanism primarily governed by the early-to-intermediate stages of deformation and the connectivity of the elements. Although the strain in the structure can be large, in the $\varepsilon_{W}$ regime the deformation reported in each element remains small enough that effects such as large displacements, nonlinear kinematics, or changes in load path due to geometry are negligible. This enables the design of uncertainty-aware, robust structures. To avoid yielding or plastic deformation in the hard elements, $E_{\mathrm{hard}}$ must be chosen so that stress remains below the elastic limit under expected loads. Overall, optimal performance emerges from a well-balanced interplay of $K_{\mathrm{CT}}^{+}$, $K_{\mathrm{CS}}^{+}$, and the $E_{\mathrm{hard}}$ of the hard platelets, ensuring robustness.

Our design remains effective as long as adhesive properties vary within a CV of roughly 25%; beyond that, interface properties can drop to unphysical values (e.g., 20% of the mean), at which point the homogenization effect breaks down. From our sensitivity analysis, we observe that when adhesive CV is below 10%, the composite’s overall modulus and strength deviate by less than 5% compared to a deterministic interface. As adhesive CV increases toward 25%, the composite still suppresses much of the input variability, but at the cost of a modest reduction in mean stiffness and strength (on the order of 10 to 15%). In practical terms, controlling adhesive properties to within 15% CV yields near-ideal homogenization; if tighter mechanical consistency is required, as in high-performance applications, keeping adhesive CV below 10% is advisable. This trade-off between interface uniformity and performance can be tuned according to end-use requirements.

In summary, our work presents a fundamental advance in creating robust structures of consistent quality from materials with inherently stochastic mechanical properties. By introducing the brick-and-mortar framework explicitly tailored for recycled polymers, the UATSC model simultaneously accommodates variability in $E_{\mathrm{hard}}$ and $\sigma_{max,CT},\tau_{max,CS},\delta_{cr,CT},\delta_{cr,CS}$. This design-driven, material-agnostic approach can be applied to any recycled polymer class without relying on chemistry-specific treatments. By applying this approach to industrial HDPE stretch film, we demonstrated 93% suppression of $E_{\mathrm{eff}}$ variability and 68% suppression of $\varepsilon_{W}$ variability compared to the input rHDPE properties, while matching the modulus of virgin stretch film. While the design achieves reliable behavior with suppressed variability, it does not match the $\varepsilon_{\max}$ of virgin HDPE films, though it exceeds that of degraded HDPE. With optimized interfaces, full 3D modeling, and film-grade processing, higher $\varepsilon_{\max}$ values are expected thus avoiding rHDPE downcycling. Moreover, through our experimental fabrication of rHDPE brick-and-mortar composites we validated the model results and showcased that such designs have the potential to be produced at scale. By focusing on designed-enabled variability suppression (instead of chemistry-specific upcycling methods, which can be challenging to scale and often pose significant environmental impacts (76), or process optimization approaches, which cannot handle widely variable feedstocks), the UATSC model offers a sustainable path to consistent performance, unlocking broader industrial use of recycled plastics and helping to address the global waste-plastic crisis. Although the “mortar” phase of the bioinspired structure requires virgin polymer, its fraction can be tuned and minimized given the desired application, thereby reducing virgin material consumption, and allowing for recycled composite structures that are both technically viable and environmentally responsible.

## Materials and Methods

### Examination of UATSC Model Performance in Limiting Cases and Model Validation.

To examine UATSC model’s performance in limiting cases and validate the model using semianalytical expressions, we analyzed its behavior under two simple scenarios: the serial chain and alternating column configurations. In the serial chain case, the system consists solely of CT and hard elements, all connected in series within a single layer. The overall $E_{\mathrm{eff}}$ decreases as the applied force from the displacement load is transmitted through all elements, with the most compliant constituents undergoing the largest deformation and dominating the structural response. The $\sigma_{\max}$ of the configuration is governed by the weakest link theory suggesting that failure occurs when the weakest constituent reaches its limit load. In the alternating column case, the network’s connectivity consists of a combination of parallel and series configurations, resulting from the alternating arrangement of platelets. $E_{\mathrm{eff}}$ is determined by the combined contribution of the CS and hard elements, with the compliant CS elements absorbing most of the deformation. Due to the complex connectivity among elements, the $\sigma_{\max}$ in this configuration does not follow the strongest-link theory—as the elements are not entirely arranged in parallel—but instead falls between the predictions of the weakest- and strongest-link models. In both cases, $E_{\mathrm{eff}}$ is heavily influenced by the properties of the softer interfaces.

To obtain an analytical expression for the $E_{\mathrm{eff}}$ of the structure for each of the two cases we analyze the connectivity of the elements. In the serial chain case, the system can be approximated as a collection of springs in series. Assuming that $K_{HARD_{i}}$ is the stiffness of the i-th hard platelet, and $K_{CT_{i}}^{+}$ is the stiffness of the i-th CT element, the overall stiffness is described by Eq. **16**,

[16] $$\frac{1}{K_{\mathrm{total}}}=\sum_{i=1}^{N}\frac{1}{{\bar{K_{\mathrm{HARD}}}}_{i}}+\sum_{i=1}^{N-1}\frac{1}{\bar{K_{CT_{i}}^{+}}},$$

where w is the width of the structure, and $\begin{array}{c} {\bar{K_{\mathrm{HARD}}}}_{i}=E_{HARD_{i}}\left(\frac{wh}{l}\right)\bar{K\overset{+}{cT_{l}}}=K_{cT_{i}} \end{array}$ are measures of stiffness with consistent units. The $\sigma_{\max}$ of the structure is equal to the minimum stress capacity observed among the CT elements, $\sigma_{\max,CT_{i}}$ (Eq. **17**).

[17] $$\sigma_{max,chain}=\min(\sigma_{max,CT_{i}}).$$

An analytical expression for $E_{\mathrm{eff}}$ in the alternating column case is less straightforward. The simplest configuration, consisting of only three layers, can be analyzed using only a combination of series and parallel spring connections. In general, for structures with an odd M, an analytical solution can be determined by extending this approach. This is possible by using the compliance C=1/K of the elements instead of K directly and considering transformations between Delta (Δ) and Wye (Y) spring connections in the network (*SI Appendix*, Fig. S5). Eventually the original network of size $\left\{N,M\right\}$ decomposes to an equivalent network of series and parallel connections. For a network consisting of five layers, the overall stiffness is given by Eq. **18**,

[18] $$K_{total}=\frac{6{\bar{C_{HARD}}}_{i}+4\bar{C_{CS_{i}}}}{5-{\bar{C_{HARD_{i}}}}^{2}+5{\bar{C_{HARD}}}_{i}\bar{C_{CS_{i}}}+{\bar{C_{CS_{i}}}}^{2}},$$

where $-\;C_{HARD_{i}}\;=1/{\bar{K_{HARD}}}_{i},\bar{C_{CS_{i}}}=1/\bar{K_{CS_{i}}^{+}},$ are the compliances of the i-th hard element and any CS element respectively. Here, $\bar{K_{CS_{i}}^{+}}=K_{CS_{i}}^{+}\left(\frac{\mathrm{wl}}{2}\right)$ is a measure of stiffness with consistent units. Due to the complex connectivity of the elements, an analytical formula for $\sigma_{\max}$ is not straightforward.

We validate our model by comparing the results obtained from Eqs. **16** to **18** with the corresponding results of the model simulations. We select rHDPE as the model material due to the availability of data compared to other recycled plastics. Assuming that $E_{\mathrm{hard}}$ follows a normal distribution, we determine its mean value from literature data (6, 8, 37–44) as $E\left[E_{\mathrm{hard}}\right]=$ 1,213 MPa with a SD of σ= 654 MPa (Sample size = 40). To ensure $E_{\mathrm{eff}}$ aligns with a reasonable normal distribution, we set σ= 200 MPa corresponding to an elevated CV of 16%. Regarding interfacial elements’ properties, $\sigma_{max,CT},\tau_{max,CS}$ are sampled from a Weibull distribution with a shape parameter m= 5.00, reflecting the high uncertainty in substrate-adhesive maximum traction, and a scale parameter s= 4.00, which is within the range reported in soft materials (75, 77). Therefore, in the validation cases, $E_{\mathrm{hard}}=N$ (1213, $200^{2}$) and $\sigma_{max,CT},\tau_{max,CS}=W\left(5.00,4.00\right)$.

Deriving an analytical expression for the distribution of the structures’ $E_{\mathrm{eff}}$ or $\sigma_{\max}$ is challenging, as the underlying distributions are not always reversible, and the interaction between both sources of variability is difficult to determine. Therefore, we use Monte Carlo simulations to assess the variability in the structure’s properties. A total of $10^{6}$ experiments are conducted for a network of size {5,1} in the serial chain configuration and {1,5} in the alternating column configuration to obtain the semianalytical distribution of $K_{\mathrm{total}}$ and $\sigma_{\max}$ (Eqs. **16**–**18**). Then $E_{\mathrm{eff}}$ is obtained through Eq. **19**.

[19] $$\sigma =\underset{{Ε}_{eff}}{\underbrace{K_{total}\left(\frac{L}{wh}\right)\frac{\Delta }{L}}}.$$

We then perform $10^{6}$ simulations using the developed computational model to monitor $E_{\mathrm{eff}}$. The model’s parameters used in the validation studies are presented in *SI Appendix*, Table S2. Here, t is set to 0.25 mm and 0.5 mm for CT and CS elements, respectively. These values are chosen to align with manufacturing constraints while ensuring compatibility with the selected platelet dimensions that match a tensile specimen of size {L,H,W}={152.5,32.5,10} using a network {N,M}={10,5}.

The distributions obtained using the semianalytical and full-model approaches closely align (*SI Appendix*, Fig. S6). The mean values match, while the variability is nearly identical in the serial chain case (*SI Appendix*, Fig. S6A) and reasonably close in the alternating column configuration (*SI Appendix*, Fig. S6B). The slight discrepancy in variability may be attributed to the form of Eq. **18** which features aggregated multiplication coefficients—whose stochasticity propagation is not exactly reflected in the full model during stiffness matrix assembly. Moreover, the model’s validity is further supported by the nature of the resulting distributions: The serial chain configuration exhibits a Weibull-like distribution, reflecting the detrimental influence of overly compliant elements on $E_{\mathrm{eff}}$ – consistent with weakest-link behavior. In contrast, the alternating column configuration yields a distribution closer to normal, emphasizing the variability-suppressing potential of increased network connectivity (details about the distributions are provided in *SI Appendix*, Fig. S7).

### Characterization of Platelet and Interface Properties.

rHDPE was used as the stiff platelet material in the bioinspired composite design. Ten commercial rHDPE sheets were sourced (Recycled Marine-Grade Moisture-Resistant Polyethylene—3762N111, McMaster-Carr, Elmhurst, IL) and cut into standardized tensile specimens following the ASTM D638 Type I dimensions (78) using a water-jet (5555 JetMachining Center, OMAX Corporation, Kent, WA) (*SI Appendix*, Fig. S8 *A* and *B*). To properly address the variability in recyclates, 10 different plates were used to manufacture the specimens (five specimens per plate). A total of 50 specimens were tested under uniaxial tension at a constant crosshead speed of 15 mm/min using a universal testing machine (Instron 5982 Electromechanical Test System, Instron, Norwood, MA) and a video extensometer (Epsilon-ONE, Epsilon Technology Corporation, South Park, WY) to monitor the elastic strain in the specimen (*SI Appendix*, Fig. S8 *C*–*F*). The selected displacement rate led to specimen failures within the suggested 8 min interval. All tests were conducted at room temperature (23 ± 1 °C) and 50% relative humidity. The stress–strain curves obtained were used to extract the tangent modulus at the point of 500 N. (*SI Appendix*, Fig. S8 *G* and *H*). The average tangent modulus was found to be 1,350 MPa, with a SD of 239 MPa, resulting in a CV of 18%. These statistics define the distribution of $E_{\mathrm{hard}}$ in the simulation framework.

PDMS was used as the soft interface binding rHDPE platelets together. To characterize the interfacial behavior, lap-shear tests were performed to extract cohesive law parameters’ values (*SI Appendix*, Fig. S9). PDMS (Sylgard 184, Dow Inc., Midland, MI) was prepared by mixing the base and curing agent in a 10:1 weight ratio and defoamed using a centrifuge machine before molding (ARE-310, THINKY, Tokyo, Japan). The rHDPE adherent strips, following the ASTM D3163 standard, were manufactured from the same as-received sheets as those used for the tensile specimens (five specimens per plate) using a waterjet (5555 JetMachining Center, OMAX Corporation, Kent, WA) (*SI Appendix*, Fig. S9 *A* and *B*). The PDMS precursor was cast between overlapping rHDPE strips (20 mm × 20 mm bonding area) using a silicone rubber mold and cured at 60 °C for 24 h. The lap-shear specimens were left to rest for more than 48 h before testing (*SI Appendix*, Fig. S9*C*). Five specimens were tested under displacement control (*SI Appendix*, Fig. S9*D*) with a constant displacement rate of 3 mm/min using a universal testing machine (Instron 68SC-1, Instron, Norwood, MA) following the ASTM D3163 (79). Interfacial failure was observed in all specimens (*SI Appendix*, Fig. S9 *E* and *F*), and the interfacial maximum traction was calculated as the peak force normalized over the bonding area (*SI Appendix*, Fig. S9*G*). The measured data were used to fit a Weibull distribution for the $\tau_{max,CS}=$ 0.25 MPa, with m= 7.00 and s= 0.20 MPa. The $\delta_{e,CS}=$ 0.80 mm parameter was considered as the mean value among the experiments while the distribution of $\delta_{cr,CS}$ was taken as $N(1.20,0.18^{2})$, based on displacement at failure. Considering the loading pattern of the network the interfacial parameters of the CT elements were chosen to be slightly weaker than those of the CS elements (*SI Appendix*, Table S3).

### Fabrication and Testing of Bio-Inspired Composite Structure.

For the stiff platelets, postconsumer recycled (PCR) plastics are typically washed, sorted, and shredded into pellets or flakes before being processed into uniform recycled plastic sheets. Cutting such sheets into platelet-sized components is both experimentally feasible and industrially scalable. In this study, platelets were cut from 10 rHDPE plates, sourced from the same batch used in tensile and lap-shear tests, ensuring consistency in material properties (*SI Appendix*, Fig. S10*A*). The rHDPE platelets were then arranged using a mold-assisted alignment process, with structural parameters guided by the UATSC model. The custom 3D-printed mold was printed with commercial PETG filament (HATCHBOX 1.75 mm PETG Filament, HATCHBOX, USA) on a PRUSA MK4 printer (Prusa Research, Prague, Czech Republic) and is shown in *SI Appendix*, Fig. S10*B*. A {8,5} network configuration was employed. The CT and CS elements were prepared by curing PDMS at a 10:1 weight ratio following the same procedure as for the lap-shear test specimens (*SI Appendix*, Fig. S10*C*). The structure’s parameters were those reported in *SI Appendix*, Table S3, leading to a total specimen length of 195 mm and height of 53 mm.

Mechanical testing of the fabricated bioinspired composite specimens was performed using a (Instron 68SC-1, Instron, Norwood, MA) equipped with a 500 N load cell. The experimental setup featured a custom in situ photoelasticity device (80) comprising a black frame and two crossed polarizers to monitor birefringence in the PDMS during loading of the bioinspired composite structure (*SI Appendix*, Fig. S10 *D*–*F*). To ensure consistency with the cohesive laws extracted from lap-shear experiments, the tensile tests were conducted at the same strain rate of 3 min^−1^ under displacement control. Specimens were gripped using flat grips to minimize damage at the gripping zone (*SI Appendix*, Fig. S10*G*). All tests were carried out at ambient laboratory conditions (23 ± 1 °C, 50% RH). Force and displacement data were recorded and used to compute the stress–strain response of each specimen. A total of five specimens were tested. The obtained photoelastic images were used to qualitatively observe the loading pattern and failure events in the structure.

## Supplementary Material

Appendix 01 (PDF)

Movie S1. Tensile experiment of the bio-inspired composite with birefringence monitoring through photoelasticity.

## Data Availability

All study data are included in the article and/or supporting information.
