# Supplementary material for: Suppressing mechanical property variability in recycled plastics via bioinspired design
Source: Proc Natl Acad Sci U S A. 2025 Aug 12;122(33):e2502613122. doi: 10.1073/pnas.2502613122 (PMC12377729; doi:10.1073/pnas.2502613122)
Supplement: Supplementary file 1 — Appendix 01 (PDF) [file pnas.2502613122.sapp.pdf]

PNAS

Supporting Information for

Suppressing Mechanical Property Variability in Recycled Plastics *via* Bio-inspired Design

Dimitrios Georgiou[1,=], Danqi Sun[1,=], Xing Liu[2], Christos E Athanasiou[1*]

1. Daniel Guggenheim School of Aerospace Engineering, Georgia Institute of Technology, Atlanta, GA 30332, USA.
2. Department of Mechanical and Industrial Engineering, New Jersey Institute of Technology, Newark, NJ 07102, USA

[=] equal contribution

Christos E. Athanasiou
Email: athanasiou@gatech.edu

This PDF file includes:

Figures S1 to S10
Tables S1 to S5
Legend for Movie S1
SI References

Other supporting materials for this manuscript include the following:

Movie S1

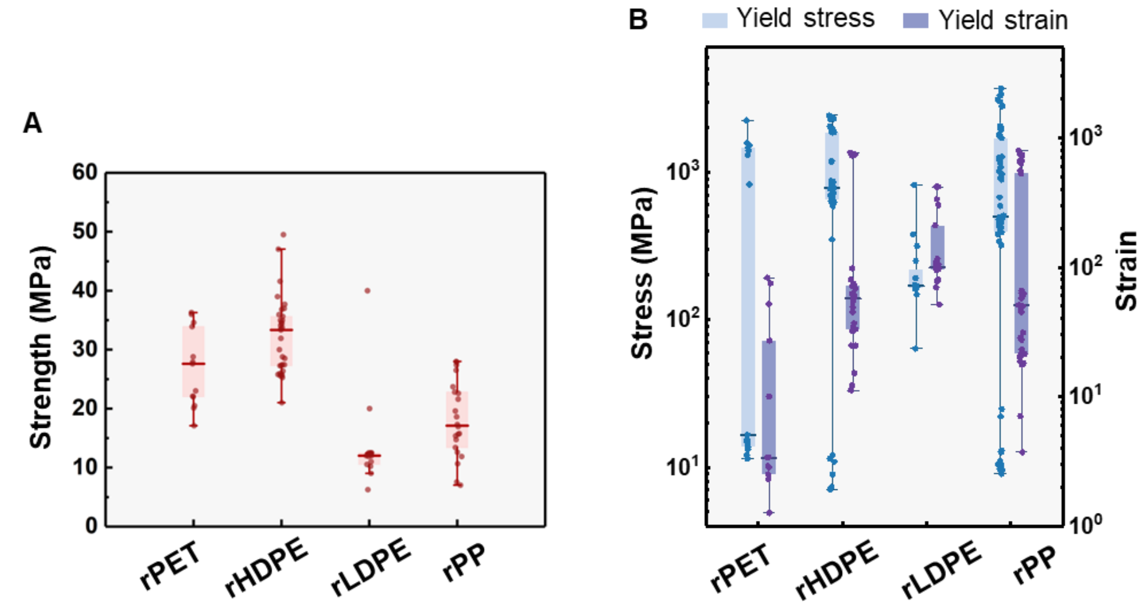

Figure S1. Literature data on the mechanical properties of recycled plastics. (A) Compilation of strength data from multiple studies, highlighting the substantial variability of recycled plastics. Outliers likely reflect differences in polymer composition, contamination, and processing history. **(B)** Yield stress and yield strain data demonstrate broad scatter across recycling conditions, attributable to differences in material blends, recycling cycles, environmental exposure, and processing parameters. The variability observed also reinforces the need for systematic experimental frameworks to better quantify and predict the mechanical behavior of recyclates. All data are drawn from references (1–11).

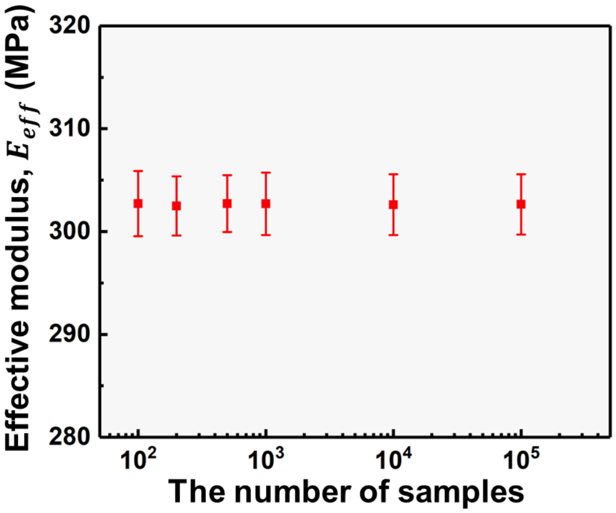

Figure S2. Convergence analysis. Convergence of the effective modulus, E_{eff}, and its variability as a function of the number of Monte Carlo simulations for a network of size $\{10,5\}$. Both the mean value (red squares) and the standard deviation (error bars) exhibit minor fluctuations, reflecting statistical confidence and indicating that 10^2 samples provide a sufficiently converged estimate of E_{eff} in both magnitude and dispersion.

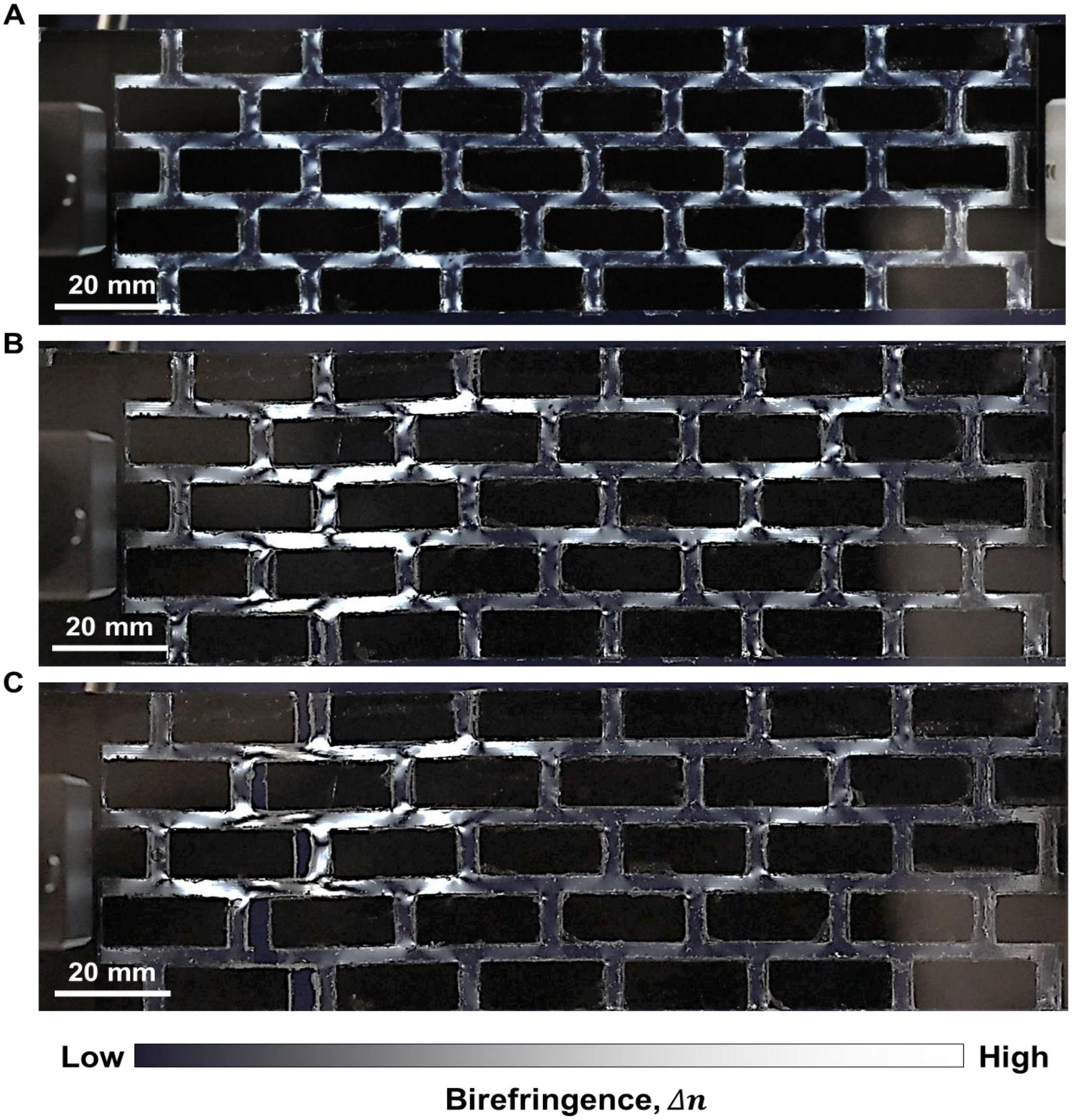

Figure S3. Assessment of contours obtained by the photoelasticity method. The three images are magnified versions of the insets ((ii) – (iv)) in Figure 5 of the main manuscript. Photoelasticity is an experimental technique that exploits stress-induced birefringence, Δn, in transparent materials. Under mechanical loading, applied stress causes variations in light velocity along two principal directions, producing birefringence patterns. These patterns allow for the measurement of principal stress differences using the stress-optic law (12). **(A)** Linear region: the structure is subjected to uniaxial loading, resulting in a uniform stress distribution within both CT and CS elements. The characteristic shear bands of the CS elements appear as bright diagonals, indicating significant shear stress. In contrast, the CT elements display lower Δn due to their lower principal stress differences (13). This lower Δn is further attributed to lower $\sigma_{max,CT}$ compared to $\tau_{max,CS}$. **(B)** Onset of damage: Initial failure occurs in some CT elements due to interfacial decohesion, and the neighboring CS elements are responsible for load transfer. Fracture of adjacent elements and connected branches or chains causes partial unloading in certain regions, leading to localized deformation. Despite this damage accumulation, the overall structure retains its load-bearing capacity. **(C)** Severe damage: all CT elements have failed, and the CS elements are extensively loaded, leading to progressive damage and eventual failure of the structure.

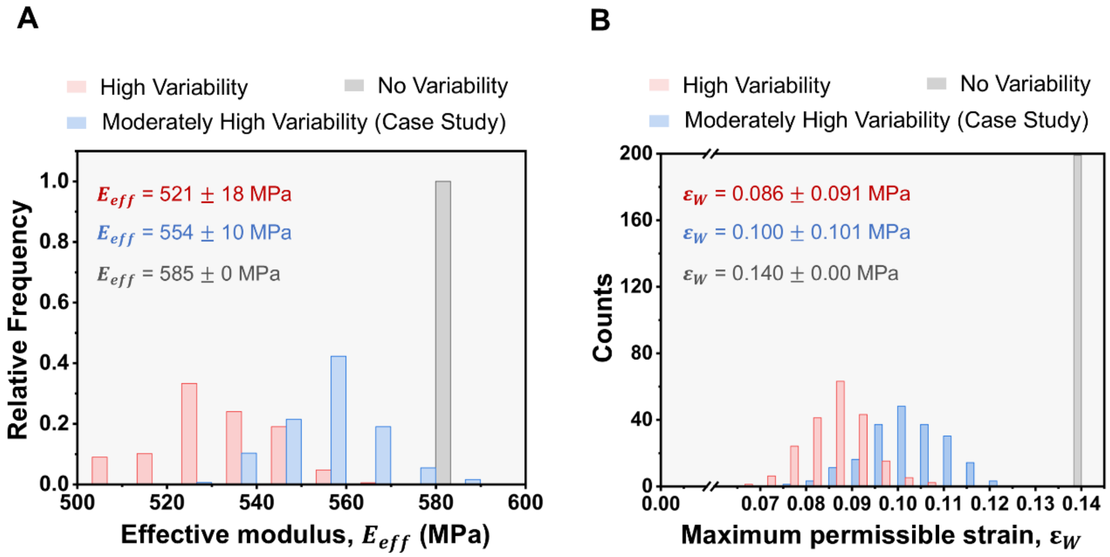

Figure S4. Effect of E_{eff} variability on the mean value of ε_W. Three cases of different variability levels for $E_{hard}, \sigma_{CT.max}, \tau_{CS.max}$ are examined. The mean value of all model parameters is the same as that reported in Table S4. The high-variability case features a 50% higher CV for E_{hard} compared to Table S4 and the shape parameters of the Weibull distribution are set to 4. The moderately-high-variability is reported in Table S4 and is identical to the one illustrated in Figure 6 of the main manuscript. The no-variability case features only deterministic properties. **(A)** Distribution of E_{eff} for the three cases. The high-variability case clearly exhibits the largest dispersion and lower E_{eff} mean value. This is due to the weakest link effect. **(B)** Distribution of ε_W for the three cases. Notably the variability in ε_W, quantified by the CV is similar in the cases featuring significant levels of variability. However, there is a clear trend of reduced ε_W with increasing E_{eff} variability, highlighting the importance of controlling E_{eff} variation.

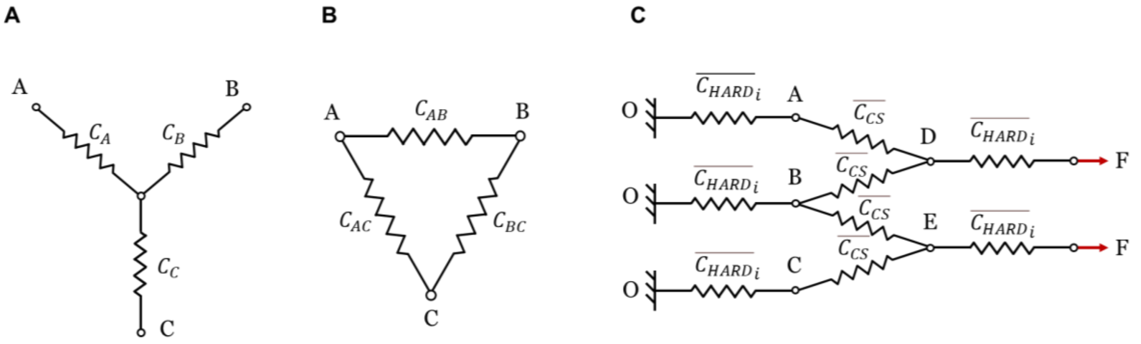

Figure S5. Analysis of the spring connectivity of the alternating column case. (A) Wye (Y) spring connectivity **(B)** Delta (Δ) spring connectivity **(C)** Connectivity of a $\{1,5\}$ alternating column network. The alternating column configuration features a mixed series-parallel connectivity that can be analyzed using these transformations. Considering the compliance C of an element, it is possible to transform a Y configuration into an equivalent Δ one and vice versa.

Y to Δ transformation:

$$C_{AB} = \frac{C_A C_B + C_B C_C + C_C C_A}{C_C}$$
$$C_{BC} = \frac{C_A C_B + C_B C_C + C_C C_A}{C_A}$$
$$C_{CA} = \frac{C_A C_B + C_B C_C + C_C C_A}{C_B}$$

Δ to Y transformation:

$$C_A = \frac{C_{AB} C_{CA}}{C_{AB} + C_{BC} + C_{CA}}$$
$$C_B = \frac{C_{AB} C_{BC}}{C_{AB} + C_{BC} + C_{CA}}$$
$$C_C = \frac{C_{BC} C_{CA}}{C_{AB} + C_{BC} + C_{CA}}$$

All fixed nodes are reduced to a single node (O), while all displacement nodes collapse into another single node (F). By systematically simplifying the network through series and parallel reductions and applying $Y - to - \Delta$ and $\Delta - to - Y$ transformations where necessary, the entangled connectivity is resolved. This process ultimately yields an equivalent stiffness value, as expressed in Eq. (15).

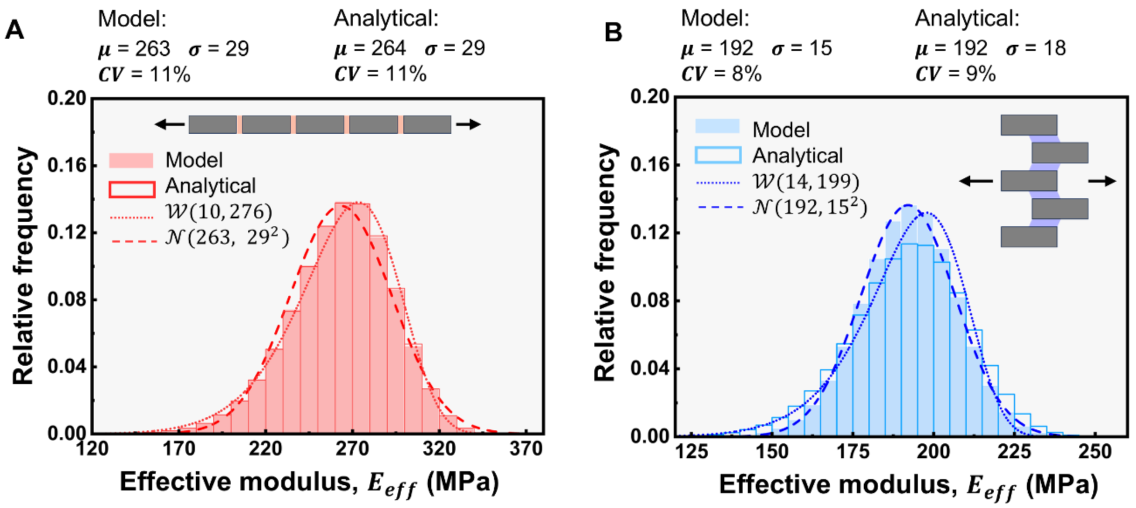

Figure S6. Examination of model performance in limiting cases. The cumulative probability distribution functions for the analytical solution are compared, in terms of mean value, μ, standard deviation σ, and CV, with the results of Monte Carlo simulations from the proposed model. **(A)** In the serial chain configuration, E_{eff} is primarily dictated by the most compliant element. The analytical predictions show excellent agreement with model simulations for μ, σ, and CV. **(B)** In the alternating column configuration, E_{eff} arises from a mix of series and parallel connections. While μ matches closely between analytical and simulation results, small discrepancies in σ and CV emerge due to the propagation of variability through the aggregated terms in the analytical solution. Together, these two limiting cases validate the predictive behavior of the model and provide insights into the variability suppression capabilities of the design.

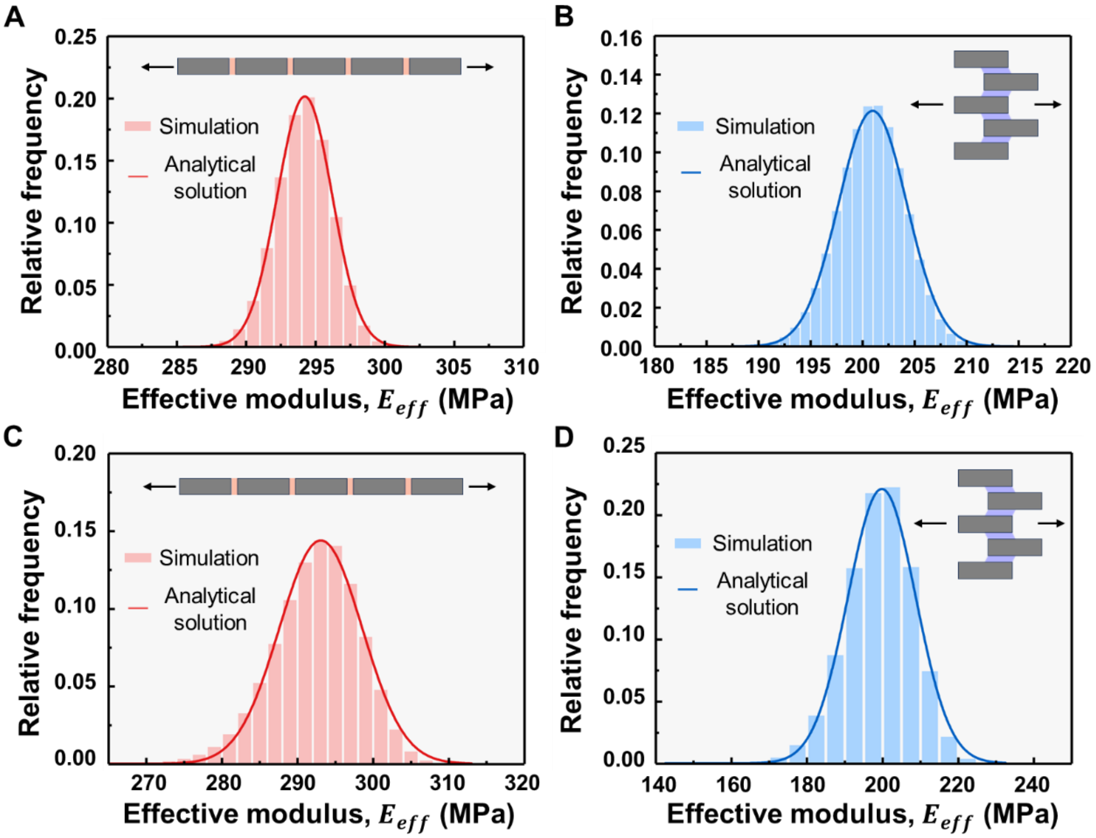

Figure S7. Effect of the magnitude of E_{hard} variability on the network's E_{eff} distribution. Distribution of the E_{eff} for low platelet variability (E_{hard} = 1213 ± 75 MPa) and deterministic interfaces for **(A)** the serial chain case, and **(B)** the alternating column case. The bottom row illustrates the distribution of the E_{eff} of the network for elevated platelet variability (E_{hard} = 1213 ± 200 MPa) and deterministic interfaces for **(C)** the serial chain case, and **(D)** the alternating column case. The effect of the variability on E_{eff} is based on the connectivity of each configuration. Increasing the variability leads to an increase in dispersion as well as a change in the profile of the distribution. In the serial chain case, where weaker constituents heavily influence the variability, a left tail in the histogram of the distribution (Figure S7A, S7C) is apparent suggesting a shift from a normal to a Weibull distribution. Although this tail is present, the histogram still fits a normal distribution more closely than a Weibull one (Table S5). Introducing uncertainty in the interfaces (Figure S6) further increases this effect as the resulting histogram fits a Weibull distribution better than a normal one (Figure S6A). In the alternating column case, where connectivity is more complex, the model's results fit a normal distribution better than a Weibull distribution despite interfacial variability (Figures S6B, S7B, S7D), indicating that connectivity plays a critical role in mitigating the adverse effects of variability. Table S5 provides metrics for the goodness of fit for the distributions shown in this figure.

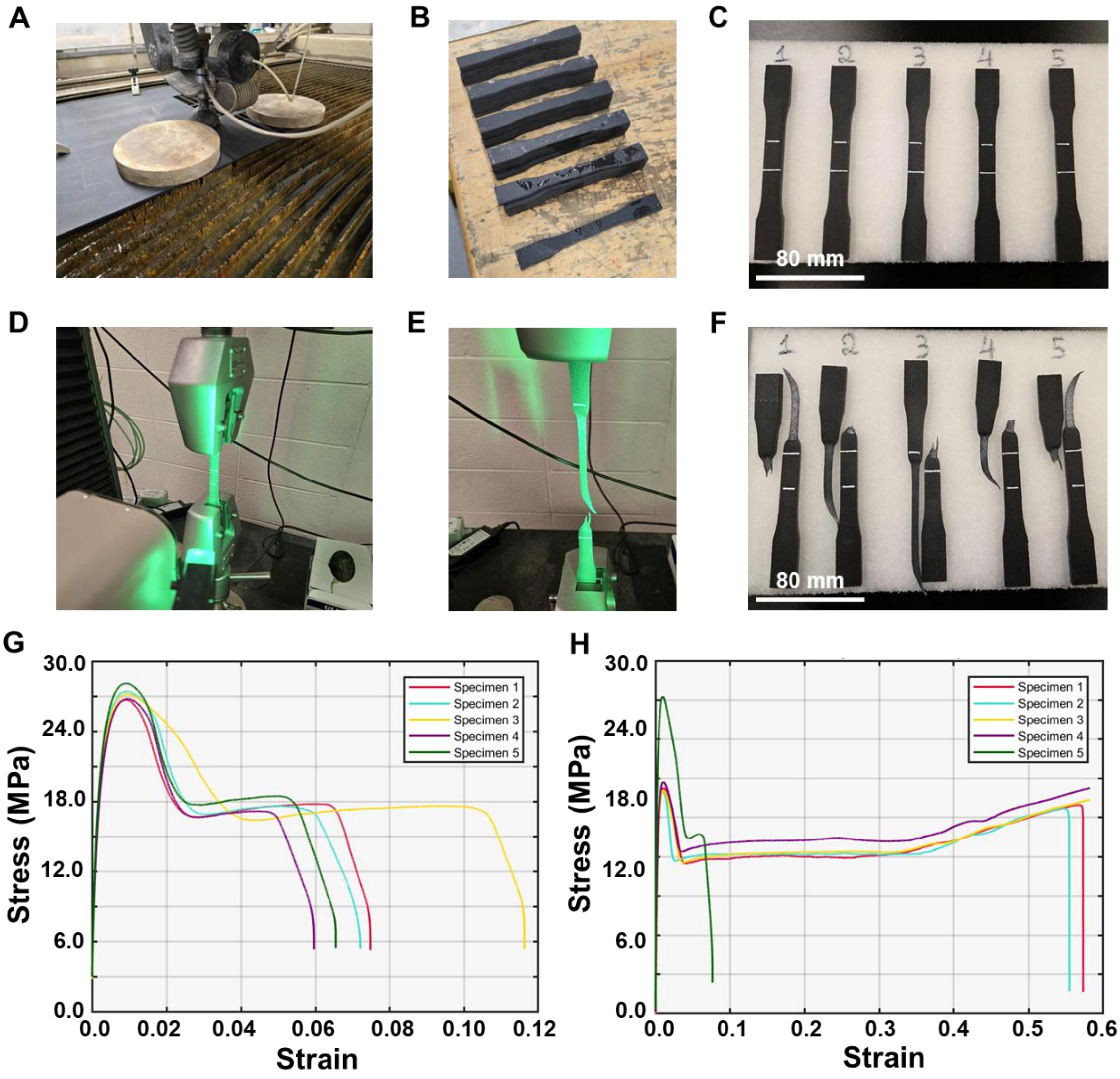

Figure S8. Process overview for mechanical testing and characterization of recycled platelets. (A) rHDPE tensile specimens cut from ten feedstock plates using a waterjet machine, following the ASTM D638 Type I standard (14). **(B)** As-cut specimens. **(C)** Specimens cleaned and dried for 96 hours, then marked for strain measurement. **(D)** Tensile testing setup (Instron 5982, 1 kN load cell, 5 mm/min crosshead speed, 23 ± 1 °C, 50% RH) with video extensometer (Epsilon One) tracking deformation up to peak stress. **(E)** Typical fracture pattern showing necking initiated by polymer chain alignment, followed by mid-section rupture. **(F)** Typical failed specimens, illustrating consistent ductile fracture. **(G, H)** Representative stress-strain curves, highlighting variation in modulus and elongation at break.

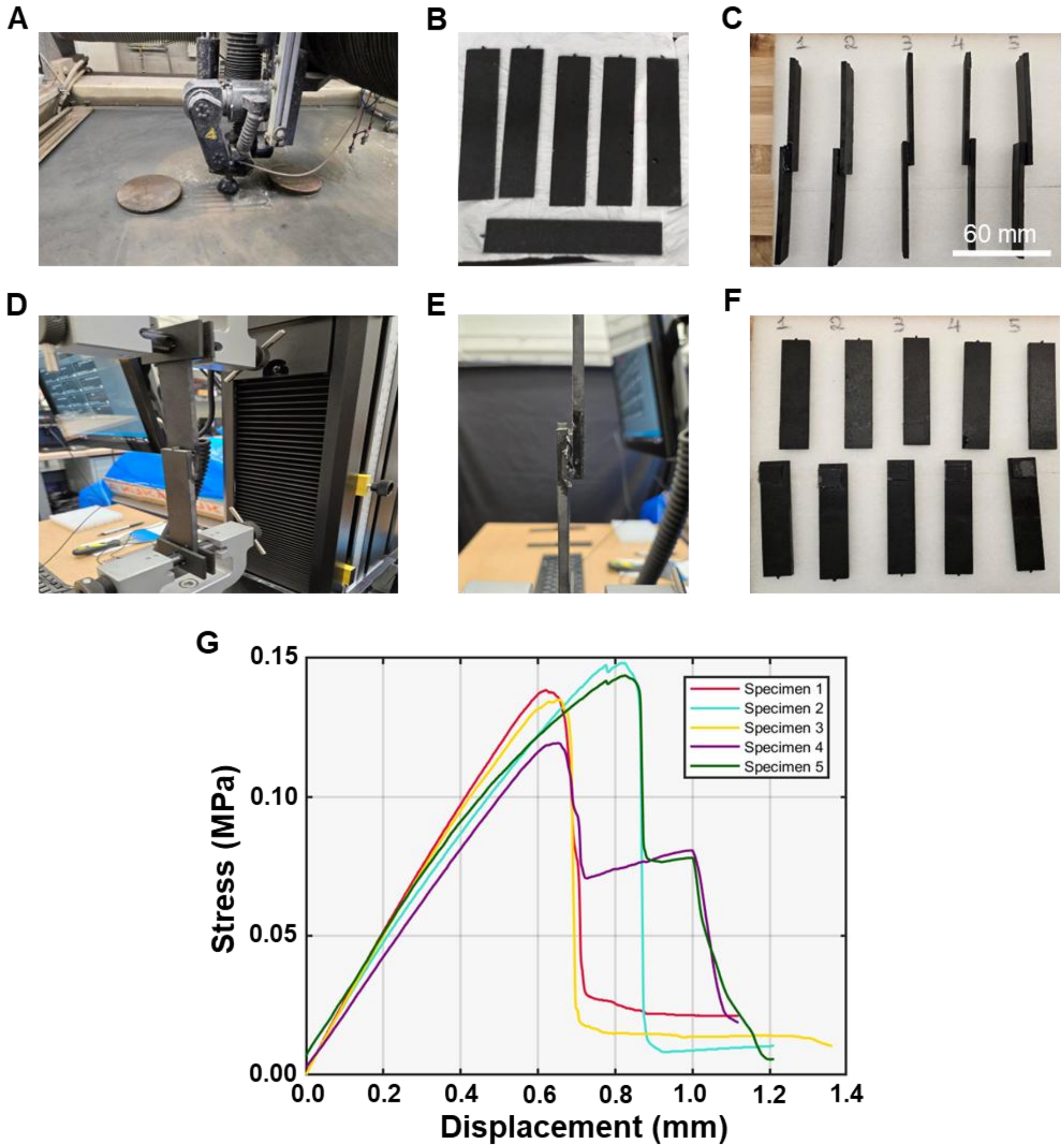

Figure S9. Process overview for interfacial material properties characterization. (A) rHDPE strips cut from ten feedstock plates using a waterjet machine, following the ASTM D3163 standard (15). **(B)** As-cut lap-shear adherends. **(C)** Lap-shear specimens are prepared by cleaning and drying strips for 96 h, casting PDMS (10:1 base:agent) into 20 mm × 20 mm overlap areas, curing for 8 h, then resting for 48 h. **(D)** Lap-shear testing setup (Instron 3365, 1 kN load cell, 1 mm/min displacement rate, 23 ±1 °C, 50% RH). **(E)** Typical failure mode. **(F)** Failed specimens displaying adhesive debonding at the PDMS–rHDPE interface. **(G)** Representative traction-separation response illustrating peak traction and critical decohesion.

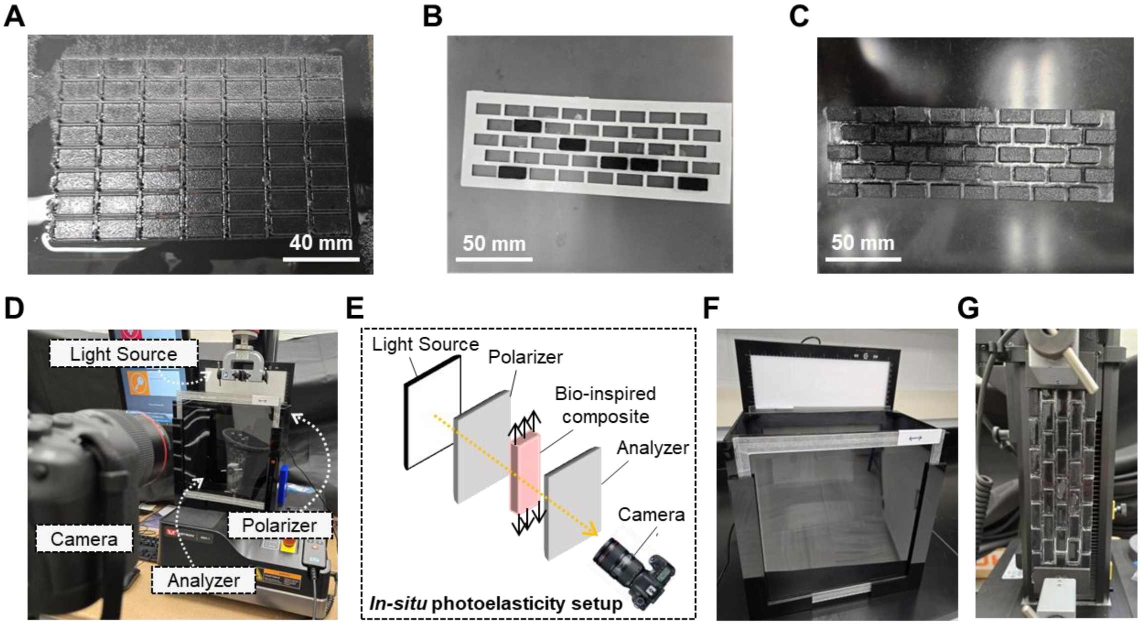

Figure S10. Bio-inspired composite fabrication and testing. (A) Recycled HDPE platelets cut from ten feedstock plates using a waterjet machine. **(B)** 3D-printed mold used for composite fabrication. Platelets are manually inserted, and PDMS is poured within a silicone rubber casing to prevent leakage. **(C)** Bio-inspired composite specimen. **(D)** In-situ photoelasticity setup integrated with a tensile testing machine, consisting of a light source, two linear polarizers, and a camera. **(E)** Schematic of the in-situ photoelasticity setup. Polarized light passes through the loaded specimen, and birefringence patterns are captured by the camera. **(F)** Close-up of the mounted setup, with polarizers aligned perpendicularly in a dark-field configuration. **(G)** Specimen gripped in the tensile frame. The gauge length is defined between the clamps.

Table S1. Previous studies on modelling of nacre-inspired structures and their variability considerations

Work	*Authors & Year*	*Main Contribution*	*Variability Considerations*
(16)	Jäger & Fratzl, 2000	Homogenized TSC-Model for staggered arrangement of mineral platelets and collagen fibrils	-
(17)	Evans et al., 2001	Identification of key parameters (plate size, aspect ratio, topological arrangement) and mechanisms (soft-phase properties) for robustness of nacre	-
(18)	Wang et. al., 2001	Combination of theory and experiments to characterize the role of asperities in the inelastic deformation of nacre	-
(19, 20)	Ji & Gao, 2003; 2004	Inclusion of shear-lag behavior, and benchmarking of the new TSC model against the FE and Mori-Tanaka methods	-
(21)	Nukala & Šimunović, 2005	Continuous-damage random-threshold fuse-network model composed of linear-elastic bonds with multiple statistically distributed failure thresholds per element, simulating progressive damage.	Linear elastic shear interfaces included random failure thresholds to model the stress-strain response
(22, 23)	Luo & Bazant, 2017; 2019	Application of normal and Weibull statistical models to strength scaling in nacre-inspired linear-elastic composites, establishing probabilistic failure predictions and accurately capturing the low-strength tail of the distribution.	Linear elastic elements included strength stochasticity to obtain the lower left tail of the failure distribution in nacre
(24)	Abid et al., 2019	Derivation of an analytical model that combines a linear-elastic hard phase with bilinear and trapezoidal shear-cohesive interfaces, to reveal how nonuniform platelet overlap drives strain localization and influences mechanical performance	The Aspect ratio of the platelets varied within the network to model its influence on the toughness of the structure
(25)	Yan et al., 2022	Integration of interfacial strength stochasticity into the TSC framework using Monte Carlo simulations to model the shear stress transfer mechanism of CS interfaces effect to the mechanisms of strain localization and crack initiation	CT and CS interfaces featured stochastic maximum traction parameters sampled with a Weibull distribution to observe crack propagation trend and the failure stress distribution.
(26)	Hunter et al., 2023	Investigation of interfacial strength stochasticity in the stress-strain response of nacre-inspired designs (two-peak vs peak-plateau-peak)	Interfacial elements sampled via a Weibull distribution to capture the transition from a two-peak to a peak-plateau-peak response

Work	*Authors & Year*	*Main Contribution*	*Variability Considerations*
This Work	Georgiou et al.	Creation of an uncertainty-aware TSC model to reveal variability suppression in nacre-inspired architectures via the interplay of deformable stochastic platelets and stochastic interfacial parameters for the design of reliable structures.	Consideration of multiple sources of stochasticity (platelet modulus, interfacial traction, and decohesion), benchmarking their cumulative effects on the structure's ensemble stiffness and strength distribution, and analyzing how network connectivity shapes the response distribution to evaluate variability suppression in nacre-inspired designs.

Table S2. Model parameters for the sensitivity analysis and limit cases validation.

Parameter	Value
E_{hard} (MPa)	$\mathcal{N}(1213,\ 200^2)$
$\{\sigma_{max,CT}, \delta_{e,CT}, \delta_{cr,CT}\}$ (MPa/mm/mm)	$\{\mathcal{W}(5.00, 4.00)$, 0.20, 0.50}
$\{\tau_{max,CS}, \delta_{e,CS}, \delta_{cr,CS}\}$ (MPa/mm/mm)	$\{\mathcal{W}(5.00, 4.00)$, 0.20, 0.50}
l (mm)	15
h (mm)	6
w (mm)	10
t (mm)	0.5

Table S3. Fabricated bio-inspired structure properties and associated model parameters.

Parameter	Value
E_{hard} (MPa)	$\mathcal{N}(1350, 239^2)$
$\{\sigma_{max,CT}, \delta_{e,CT}, \delta_{cr,CT}\}$ (MPa/mm/mm)	$\{\mathcal{W}(7.00, 0.15)$, 0.80, $\mathcal{N}(1.20, 0.18^2)\}$
$\{\tau_{max,CS}, \delta_{e,CS}, \delta_{cr,CS}\}$ (MPa/mm/mm)	$\{\mathcal{W}(7.00, 0.20)$, 0.40, $\mathcal{N}(0.80, 0.12^2)\}$
l (mm)	20
h (mm)	8
w (mm)	4.75
t (mm)	3

Table S4. Model parameters for the case study.

Parameter	Value
E_{hard} (MPa)	$\mathcal{N}(1350, 239^2)$
$\{\sigma_{max,CT}, \delta_{e,CT}, \delta_{cr,CT}\}$ (MPa/mm/mm)	$\{\mathcal{W}(7.00, 8.00)$, 0.15, $\mathcal{N}(1.00, 0.10^2)\}$
$\{\tau_{max,CS}, \delta_{e,CS}, \delta_{cr,CS}\}$ (MPa/mm/mm)	$\{\mathcal{W}(7.00, 8.00)$, 1.40, $\mathcal{N}(1.80, 0.18^2)\}$
l (mm)	25
h (mm)	3
w (mm)	1
t (mm)	1

Table S5. Comparison of distribution properties between the material, the analytical solution, and the model for the validation configurations. The skewness values indicate that both configurations exhibit left-skewed distributions, meaning that weaker elements disproportionately affect the overall stiffness, a trend more pronounced in the serial chain model. This asymmetry is further reflected in the excess kurtosis, which suggests that the serial chain configuration has a sharper peak and heavier tails than the alternating column case, indicating a higher likelihood of extreme values. The goodness of fit metrics (R^2) for the normal and Weibull distributions reveal that E_{eff} follows a near-normal distribution, with slightly better fits for the normal distribution in both configurations, validating the statistical approach used in this analysis.

		**Low variability case (Figs. S7A & S7B)**				**High variability case (Figs. S7C & S7D)**			
		**Serial chain**		**Alternating column**		**Serial chain**		**Alternating column**	
**Metric**	**Material**	**Analytical**	**Model**	**Analytical**	**Model**	**Analytical**	**Model**	**Analytical**	**Model**
Mean μ	1213	294	294	201	201	292	292	199	199
Std. σ	200.00	1.96	1.96	3.25	3.14	5.67	5.67	9.04	8.74
CV	16%	1%	1%	2%	2%	2%	2%	5%	4%
Skewness	0	-0.1270	-0.1270	-0.0973	-0.0989	-0.4490	-0.4420	-0.2980	-0.3070
Excess kurtosis	0	0.0266	0.0266	0.0283	0.0283	0.5075	0.4895	0.2130	0.2380
R^2 value ($\mathcal{N}$)	1.0000	0.9998	0.9998	0.9999	0.9999	0.9875	0.9875	0.9940	0.9940
R^2 value ($\mathcal{W}$)	N/A	0.8412	0.8412	0.7753	0.7731	0.9789	0.9767	0.9720	0.9730

Movie S1 (separate file). Tensile experiment of the bio-inspired composite with birefringence monitoring through photoelasticity.

SI References

1. C. Pattanakul, S. Selke, C. Lai, J. Miltz, Properties of recycled high density polyethylene from milk bottles. *J Appl Polym Sci* **43**, 2147–2150 (1991).
2. F. P. La Mantia, M. Vinci, Recycling poly(ethyleneterephthalate). *Polym Degrad Stab* **45**, 121–125 (1994).
3. A. Boldizar, A. Jansson, T. Gevert, K. Möller, Simulated recycling of post-consumer high density polyethylene material. *Polym Degrad Stab* **68**, 317–319 (2000).
4. H. M. da Costa, V. D. Ramos, M. G. de Oliveira, Degradation of polypropylene (PP) during multiple extrusions: Thermal analysis, mechanical properties and analysis of variance. *Polym Test* **26**, 676–684 (2007).
5. P. Brachet, L. T. Høydal, E. L. Hinrichsen, F. Melum, Modification of mechanical properties of recycled polypropylene from post-consumer containers. *Waste Management* **28**, 2456–2464 (2008).
6. A. A. Mendes, A. M. Cunha, C. A. Bernardo, Study of the degradation mechanisms of polyethylene during reprocessing. *Polym Degrad Stab* **96**, 1125–1133 (2011).
7. H. Jin, J. Gonzalez-Gutierrez, P. Oblak, B. Zupančič, I. Emri, The effect of extensive mechanical recycling on the properties of low density polyethylene. *Polym Degrad Stab* **97**, 2262–2272 (2012).
8. P. Oblak, J. Gonzalez-Gutierrez, B. Zupančič, A. Aulova, I. Emri, Processability and mechanical properties of extensively recycled high density polyethylene. *Polym Degrad Stab* **114**, 133–145 (2015).
9. J. Aurrekoetxea, M. A. Sarrionandia, I. Urrutibeascoa, M. Ll. Maspoch, Effects of recycling on the microstructure and the mechanical properties of isotactic polypropylene. *J Mater Sci* **36**, 2607–2613 (2001).
10. M. K. Eriksen, J. D. Christiansen, A. E. Daugaard, T. F. Astrup, Closing the loop for PET, PE and PP waste from households: Influence of material properties and product design for plastic recycling. *Waste Management* **96**, 75–85 (2019).
11. V. S. Cecon, G. W. Curtzwiler, K. L. Vorst, Evaluation of mixed #3–7 plastic waste from material recovery facilities (MRFs) in the United States. *Waste Management* **171**, 313–323 (2023).
12. E. G. Coker, Filon L.N.G., Jessop H.T., *A treatise on photoelasticity*, 2nd Ed. (Cambridge U.P., 1957).
13. D. Sun, T. Lu, T. Wang, Nonlinear photoelasticity of rubber-like soft materials: comparison between theory and experiment. *Soft Matter* **17**, 4998–5005 (2021).
14. "ASTM Standard D638, 2014, 'Standard Test Method for Tensile Properties of Plastics,' ASTM International, West Conshohocken, PA, 2014, DOI: 10.1520/D0638-14, www.astm.org."
15. "ASTM Standard D3163, 2014, 'Standard Test Method for Determining Strength of Adhesively Bonded Rigid Plastic Lap-Shear Joints in Shear by Tension Loading', ASTM International, West Conshohocken, PA, 2014, DOI: 10.1520/D3163-01R14, www.astm.org."
16. I. Jäger, P. Fratzl, Mineralized collagen fibrils: A mechanical model with a staggered arrangement of mineral particles. *Biophys J* **79**, 1737–1746 (2000).
17. A. G. Evans, *et al.*, Model for the robust mechanical behavior of nacre. *J Mater Res* **16**, 2475–2484 (2001).
18. R. Z. Wang, Z. Suo, A. G. Evans, N. Yao, I. A. Aksay, Deformation mechanisms in nacre. *J Mater Res* **16**, 2485–2493 (2001).
19. H. Gao, B. Ji, I. L. Jäger, E. Arzt, P. Fratzl, Materials become insensitive to flaws at nanoscale: Lessons from nature. *Proceedings of the National Academy of Sciences* **100**, 5597–5600 (2003).
20. B. Ji, H. Gao, Mechanical properties of nanostructure of biological materials. *J Mech Phys Solids* **52**, 1963–1990 (2004).
21. P. K. V. V. Nukala, S. Šimunović, Statistical physics models for nacre fracture simulation. *Phys Rev E Stat Nonlin Soft Matter Phys* **72** (2005).

22. W. Luo, Z. P. Bažant, Fishnet model for failure probability tail of nacre-like imbricated lamellar materials. *Proc Natl Acad Sci U S A* **114**, 12900–12905 (2017).
23. W. Luo, Z. P. Bažant, Fishnet statistical size effect on strength of materials with nacreous microstructure. *Journal of Applied Mechanics, Transactions ASME* **86** (2019).
24. N. Abid, J. W. Pro, F. Barthelat, Fracture mechanics of nacre-like materials using discrete-element models: Effects of microstructure, interfaces and randomness. *J Mech Phys Solids* **124**, 350–365 (2019).
25. Y. Yan, Z. L. Zhao, X. Q. Feng, H. Gao, Nacre's brick–mortar structure suppresses the adverse effect of microstructural randomness. *J Mech Phys Solids* **159** (2022).
26. G. Hunter, L. Djumas, A. Molotnikov, L. Brassart, Modelling the effect of layer strength distribution on the brick-and-mortar failure regimes and properties. *Mechanics of Materials* **187**, 104820 (2023).
